# Supplementary material for: A vascularized breast cancer spheroid platform for the ranked evaluation of tumor microenvironment-targeted drugs by light sheet fluorescence microscopy
Source: Nat Commun. 2024 Apr 27;15:3599. doi: 10.1038/s41467-024-48010-z (PMC11055956; doi:10.1038/s41467-024-48010-z)
Supplement: Supplementary file 1 — Supplementary Information [file 41467_2024_48010_MOESM1_ESM.pdf]

A vascularized breast cancer spheroid platform for the ranked evaluation of tumor microenvironment-targeted drugs by light sheet fluorescence microscopy

David Ascheid[1 #], Magdalena Baumann[1#], Jürgen Pinnecker[2], Mike Friedrich[2], Daniel Szi-Marton[1], Cornelia Medved[1], Maja Bundalo[3], Vanessa Ortmann[1], Asli Öztürk[1], Rajender Nandigama[1,4], Katherina Hemmen[2], Süleymann Ergün[1], Alma Zernecke[3], Matthias Hirth[5], Katrin G. Heinze[2*] and Erik Henke[1,6*]

- Supplementary Information -

[1] Institute of Anatomy and Cell Biology, Universität Würzburg, Würzburg, Germany

[2] Chair of Molecular Microscopy, Rudolf-Virchow-Center for Integrative and Translational Bioimaging, Universität Würzburg, Würzburg, Germany.

[3] Institute of Experimental Biomedicine, Universitätsklinikum Würzburg, Würzburg, Germany

[4] Max Planck Institute of Heart and Lung Research, Bad Nauheim, Germany

[5] Institut für Medientechnik, Technische Universität Illmenau, Illmenau, Germany

[6] Graduate School for Life Sciences, Universität Würzburg, Würzburg, Germany

[#] These authors contributed equally to this work

[*] To whom correspondence should be addressed:

Katrin Heinze, PhD

Rudolf-Virchow-Zentrum für Experimentelle Biomedizin

Universität Würzburg

Josef-Schneider-Straße 2

97080 Würzburg, Germany.

katrin.heinze@virchow.uni-wuerzburg.de

Tel: +49-(0)931-3184214

Erik Henke, PhD

Institute of Anatomy and Cell Biology

Universität Würzburg

Koellikerstrasse 6

97070 Würzburg, Germany

Email: erik.henke@uni-wuerzburg.de

Tel: +49-(0)931-318327

Supplementary Table 1: Expression status and molecular subtypes of cell lines used[1)]

Cell line	Her2 status	ER/PR status	CK5/6 Status	EGFR status	Subtype
MDA-MB-231	-/+	-	-	+	5NP
MDA-MB468	-	-	-	+++	5NP
MDA-MB-435s[2)]	+++	-	-	-	NA
SK-BR-3	+++	-	-	++	Her2
MCF7	-	+	-	+	Luminal A
ZR-75-1	++	+	-	+	Luminal A

[1)] Expression status according to Subik *et al.* [1]. Subtypes according to [2]. 5NP: five-marker negative phenotype.

[2)] MDA-MB-435s is a melanoma line (see [3]).

ER: estrogen receptor; PR: progesterone receptor; CK: cytokeratin.

Supplementary Table 2: Staining and embedding procedure for VTSs

	Step	Solution/Solvent	Duration	Temp.
**Fixation**	Washing	PBS	-	r.t.
	Fixation	4% PFA in PBS	1h	r.t.
	Dehydration/Postfixation	50% MeOH	20 min	r.t.
		80% MeOH	20 min	r.t.
		100% MeOH	10 min	r.t.
**Storage**		100% MeOH	Up to several months	-20°C
**Preparation for staining**	Rehydration	20% DMSO in MeOH	20 min	r.t.
		20% DMSO in MeOH	20 min	r.t.
		80% MeOH	10 min	r.t.
		50% MeOH	10 min	r.t.
		PBS	20 min	r.t.
		PBS	20 min	r.t.
	Increase AB permeability	Penetration buffer	1h	r.t.
	Blocking of unspecific AB binding<br>Blocking with low-fat milk has been shown to yield better staining of ECM proteins (e.g. Col IV)	Blocking buffer<br>or<br>5% low-fat milk in PBS	1h	r.t.
**Staining**	1° AB application	1° ABs in antibody buffer	ON	4°C
	Washing	Washing buffer	6 x 30 min	r.t.
	2° AB application	2° ABs in antibody buffer	ON	4°C
	Optional:<br>DAPI counterstaining	DAPI 1 nM in PBS	4 h	r.t.
	Washing	Washing buffer	8 x 20 min	r.t.
**Embedding**	Washing	10 mM TrisHCl pH 9.0	2 x 3 min	r.t.
	Warming	-	10 min	55°C
	Embedding	100 µL/MRV 1.2% agarose in water	-	55°C
	Hydrogel setting	-	1 h	r.T
**Clearing**	Dehydration	50% EtOH in 10 mM TrisHCl pH 9.0	1h	r.t.
		70% EtOH in 10 mM TrisHCl pH 9.0	1h	r.t.
		90% EtOH in 10 mM TrisHCl pH 9.0	1h	r.t.
		96% EtOH /technical grade)	2 x 1h	r.t.
		$EtOH_{abs}$	4h	r.t.
		$EtOH_{abs}$	ON	r.t.
	Clearing	Ethyl cinnamate	4h	rt
		Ethyl cinnamate	ON → Storage until imaging	rt

Supplementary Table 3: Descriptive parameters of VTSs gained from 3D-structure analysis

**Compartmentalization**					
**CD31 Segmentation**	**Fibroblast Segmentation**	**Tumor Cell Segmentation**	**Col IV Segmentation**	**Macrophage Segmentation**	**VTS Segmentation**
CD31 Surf. Area Ave.	Fibroblasts Surf. Area Ave.	Tumor Cell Surf. Area Ave.	Col IV Surf. Area Ave.	Macrophage Surf. Area Ave.	VTS Surf. Area
CD31 Surf. Area Sum	Fibroblasts Surf. Area Sum	Tumor Cell Surf. Area Sum	Col Iv Surf. Area Sum	Macrophage Surf. Area Sum	VTS Sphericity
CD31 Sphericity Ave.	Fibroblasts Sphericity Ave.	Tumor Cell Sphericity Ave.	Col IV Sphericity Ave.	Macrophage Vol. Sum	VTS Vol.
CD31 Vol. Sum	Fibroblasts Vol. Sum	Tumor Cell Vol. Sum	Col IV Vol. Sum	% Macrophage Vol.	
CD31 Vol. Ave.	Fibroblasts Vol. Ave.	Tumor Cell Vol. Ave.	Col IV Vol. Ave.		
% CD31 Vol.	% Fibroblast Vol.	% Tumor Cell Vol.	% Col IV Vol.		

**Tracing**
**CD31 Tracing**
PV Networks Number
PV Network Surf. Area Ave.
PV Network Surf. Area Sum
PV Network Ave. Segm.
PV Network Length Ave.
PV Network Vol. Ave.
PV Network Vol. Sum
PV Segm. Surf. Area Ave.
PV Segm. Surf. Area Sum
PV Segm. Branch Level
PV Segm. Length Ave.
PV Segm. Length Sum
PV Segm. Mean Diameter
PV Segm. Straightness
PV Segm. Vol. Ave.
PV Segm. Number
PV Segm. Branching Angle

**Cell identification**		
**Tumor Cell**	**Fibroblasts**	**Macrophages**
Tumor Cell-Tumor Cell Dist. next Neighbor	Fibroblasts-Fibroblasts Dist. next Neighbor	Macrophage - Macrophage Dist. Next Neighbor
Tumor Cell-Tumor Cell Dist. nine Neighbors	Fibroblasts-Fibroblasts Dist. nine Neighbors	Macrophage - Macrophage Dist. nine Neighbors
Tumor Cells Density in VTS	Fibroblast Density in VTS	Macrophage Density in VTS
Tumor Cell-Fibroblast Dist.	Fibroblast-Tumor Cell Dist.	Macrophage-Fibroblast Dist.
Tumor Cell-Macrophage Dist.	Fibroblast-Macrophage Dist.	Macrophage-Tumor Cell Dist.
Tumor Cell-PV Dist.	Fibroblast-PV Dist.	Macrophage-PV Dist.
Tumor Cell-VTS Surf. Dist.	Fibroblast-VTS Surf. Dist.	Macrophage-VTS Surf. Dist.

**Dist. Transformation**
**CD31 Dist. Transformation**
PV Heterogeneity Index
PV Supply Index

Supplementary Table 4: List of inhibitors

Structure	Name	Function	Ref.
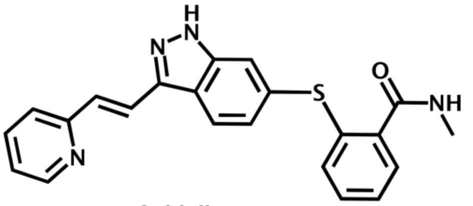<br>Axitinib	**Axitinib (AXI)**<br><br>(AG-013736)<br><br>Inlyta, Axinix<br><br>N-methyl-2-[[3-[(E)-2-pyridin-2-ylethenyl]-1H-indazol-6-yl]sulfanyl]benzamide<br><br>CAS: 319480-85-0	• VEGF-R1, -R2, -R3 inhibitor<br>• PDGF-Rβ inhibitor<br>• c-Kit inhibitor	4, 5
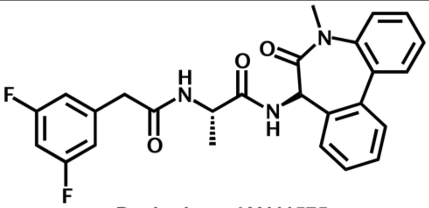<br>Deshydroxy LY411575	**Deshydroxy LY-411575**<br><br>(S,S)-2-[2-(3,5-Difluorophenyl)acetylamino]-N-(5-methyl-6-oxo-6,7-dihydro-5H-dibenzo[b,d]azepin-7-yl)propionamide<br><br>CAS-No: 209984-56-5	• γ-Secretase inhibitor	6
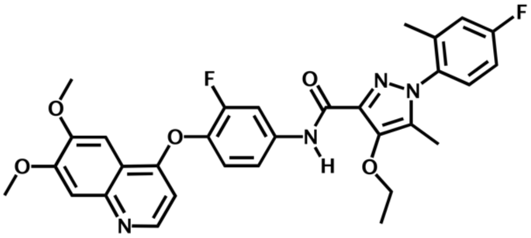<br>LDC1267	**LDC1267**<br><br>N-[4-(6,7-dimethoxyquinolin-4-yl)oxy-3-fluorophenyl]-4-ethoxy-1-(4-fluoro-2-methylphenyl)pyrazole-3-carboxamide<br><br>Cas-No: 1361030-48-9	• Mer-, Tyro3-, Axl-inhibitor (pan-TAM-RTK inhibitor)	7
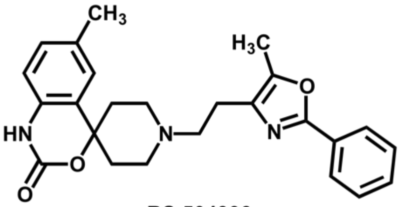<br>RS 504393	**RS-504393**<br><br>6-methyl-1'-[2-(5-methyl-2-phenyl-1,3-oxazol-4-yl)ethyl]spiro[1*H*-3,1-benzoxazine-4,4'-piperidine]-2-one<br><br>Cas-No: 300816-15-3	• CCR2 Inhibitor	8, 9
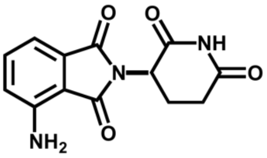<br>Pomalidomide	**Pomalidomide**<br><br>Imnovid, Pomalyst<br><br>4-amino-2-(2,6-dioxopiperidin-3-yl)isoindole-1,3-dione<br><br>Cas-No: 19171-19-8	• Immune-modulatory<br>• TNFα-antagonist<br>• IFNγ, IL6, IL10 agonist<br>• Anti-angiogenic	10, 11

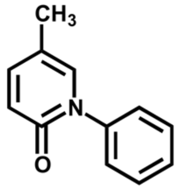 Pirfenidone	**Pirfenidone**<br><br>5-methyl-1-phenylpyridin-2-one<br><br>Cas-No: 53179-13-8	• Antifibrotic<br>• TGF-signaling antagonist<br>• TNFα-antagonist<br>• Inhibits fibroblast proliferation	12, 13
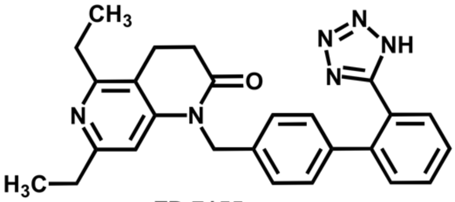 ZD 7155	**ZD 7155**<br><br>5,7-Diethyl-3,4-dihydro-1-[[2'-(1H-tetrazol-5-yl)[1,1'-biphenyl]-4-yl]methyl]-1,6-naphthyridin-2(1H)-one hydrochloride<br><br>Cas-No: 146709-78-06	• Angiotensin II inhibitor<br>• Inhibits collagen production by fibroblasts	14, 15
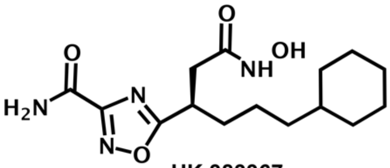 UK 383367	**UK 383367**<br><br>βR)-3-(aminocarbonyl)-β-(3-cyclohexylpropyl)-N-hydroxy-1,2,4-oxadiazole-5-propanamide<br><br>Cas-No: 348622-88-8	• BMP1 inhibitor	16, 17
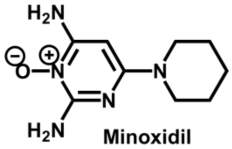 Minoxidil	**Minoxidil**<br><br>3-hydroxy-2-imino-6-piperidin-1-ylpyrimidin-4-amine<br><br>Cas-No: 38304-91-5	• PLOD2-inhibitor<br>• K^+-channel agonist	18-20
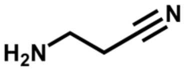 2-Amino propionitrile	**βAPN**<br><br>2-amino propionitrile<br><br>Cas-No: 2134-48-7	• Lysyl oxidase inhibitor	21-23

Supplementary Table 5: Dilution of antibodies for *in situ* staining of VTSs

Antigen	Antibody	Stock concentration	Working dilution in staining buffer
CD31	Dako, Mouse, Cat# M0832, RRID AB_2114471	0.2 mg/mL	1:100
CD11b	SigmaAldrich, Rabbit, Cat# SAB5600105, RRID:AB_2910138	2.4 mg/mL	1:80
Collagen IV	Bio-Rad, Rabbit, Cat# 2150-1470, RRID:AB_2082660	Proprietary	1:50
Hif1α	Bethyl, Rabbit, Cat# A300-286A, RRID:AB_2117114	0.2 mg/mL	1:50
Cleaved-Caspase 3	Cell Signaling, Cat# 9661, RRID:AB_2341188	Proprietary	1:50
goat anti-mouse-Cy5	Jackson ImmunoResearch, Cat# 115-175-166, RRID:AB_2338714	0.5 mg/mL	1:300
goat anti-rabbit-AlexaFluor 750	Thermofisher, Cat# A-21039, RRID:AB_2535710	2 mg/mL	1:500

Supplementary Table 6: Exemplary parameters for surface segmentation in Imaris

General
Enable Region Of Interest = false
Enable Region Growing = false
Enable Tracking = false
Enable Shortest Distance = true
Source Channel
Source Channel Index = 1-5 *(Dependent on analyzed compartment)*
Enable Smooth = true
Surface Grain Size = 1.00 µm *(10 µm for VTS generation)*
Enable Eliminate Background = true
Diameter Of Largest Sphere = 7.50 µm
Thresholding
Enable Automatic Threshold = true *(first an optimal threshold was determined using the automatic threshold function. This threshold was subsequently used for the generation of surfaces within the whole data set).*
Active Threshold = true
Enable Automatic Threshold B = false
Active Threshold B = false
Filtering
"Number of Voxels Img=1" above 200 (1e7 for VTS generation)

Supplementary Table 7: Parameters for tracing of pseudovessels in Imaris

General
Name = Autopath (loops)
Segment Start Point = false
Detect Spines = false
Enable Regions of Interest = false
Track (over time) = false
Seed Points for Segments
Segment Channel Index = 1
Segment Seed Point Diameter = 2.00 µm
Segment Seed Point Diameter Max = 30.0 µm
Filter Seed Points for Segments
Segment Seed Point Threshold = automatic *(first an optimal threshold was determined using the automatic threshold function. This threshold was subsequently used for the generation of surfaces within the whole data set).*
Diameter around Starting Point(s) to remove Seed Points = 50.0 µm
Segment Diameter Filter Strengthness = 2
Segment Classification
Group Name = Filter
Input = All Segment
No. of Classes = 2
Class:: Name = Keep
Color = 0.000 1.000 1.000
Class:: Name = Discard
Color = 1.000 0.000 0.000
FilterType = ML
Training Data
Class:: Name = Keep
Size = 50
Class:: Name = Discard
Size = 50
Max Gap Length = 0
Terminal Segment Postfilter
"Length" below 10.0

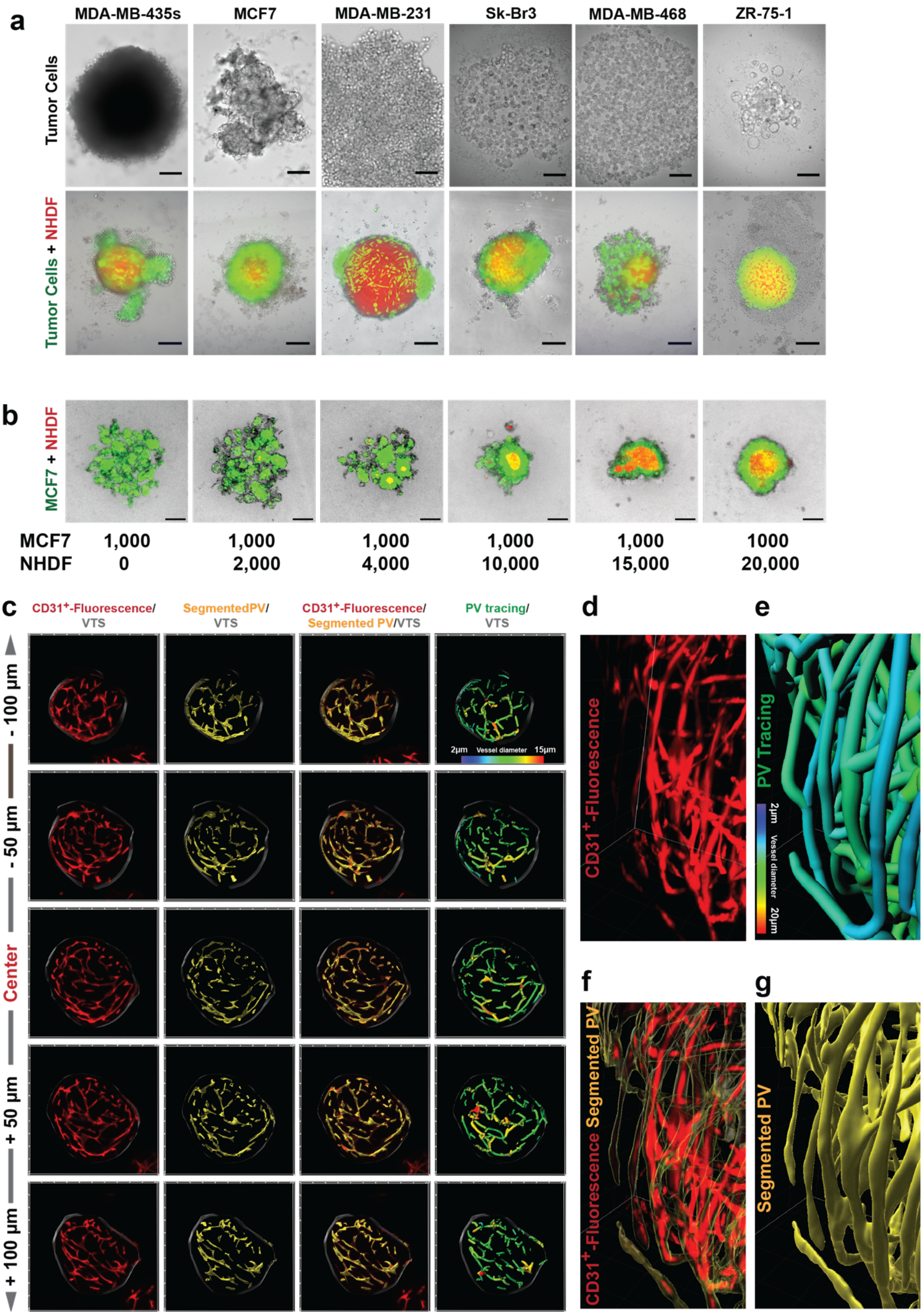
a
MDA-MB-435s
MCF7
MDA-MB-231
Sk-Br3
MDA-MB-468
ZR-75-1
Tumor Cells
Tumor Cells + NHDF
b
MCF7 + NHDF
MCF7 1,000 1,000 1,000 1,000 1,000 1000
NHDF 0 2,000 4,000 10,000 15,000 20,000
c
CD31+-Fluorescence/ VTS
SegmentedPV/ VTS
CD31+-Fluorescence/ Segmented PV/VTS
PV tracing/ VTS
- 100 µm
- 50 µm
Center
+ 50 µm
+ 100 µm
2µm Vessel diameter 15µm
d
CD31+-Fluorescence
e
PV Tracing
2µm Vessel diameter 20µm
f
CD31+-Fluorescence Segmented PV
g
Segmented PV

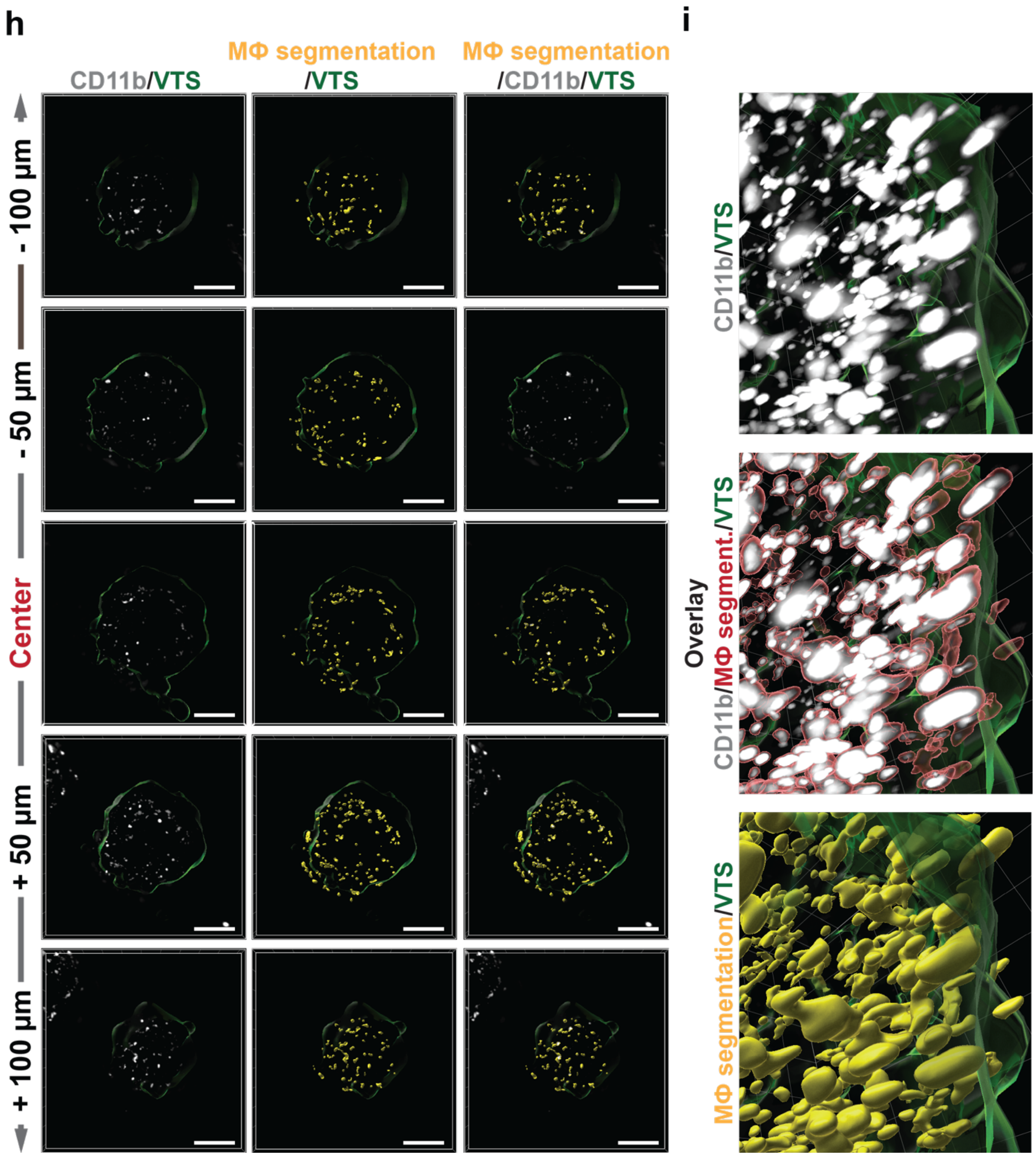

Supplementary Figure 1: VTS cultivation and 3D evaluation

a Tumor cells cultivated alone or together with NHDF in agarose-coated 96-well MWD. Upper row: From six tumor cell lines cultivated using the liquid overlay technique without the addition of NHDF only one line (MDA-MB-435s) formed solid MCTS. Lower row: Co-cultivation with NHDF enabled the formation of spheroidal aggregates from all six cell lines. Tumor cells were labeled by expression of GFP (green) and NHDF by DsRed (red) allowing for the evaluation of differences in tumor cell distribution in VTSs generated with different cell lines. **b** Results

from co-cultivation experiments with 2,000 MCF7 (green) with NHDF (red) in different ratios. Only with a surplus of NHDF VTSs were formed reliably. **c** Segmentation and tracing of PVs in a ZR-75-1-based VTS. The pre-filtered fluorescence signal from CD31-staining is shown in red, the resulting segmented and rendered PVs are in solid yellow. Displayed are also the traced and color-coded (according to mean diameter) PVs, from the same FOV. Series of five frontal cuts in the xy-plane through VTS center, and at -100 µm, -50 µm, 50 µm and 100 µm from the center in the z-direction. Depth of each cut: 20 µm. **d** Pre-filtered fluorescence signal from CD31-staining (red) in a ZR-75-1-based VTS. **e** Color-coded (according to mean diameter) PVs traced from CD31-fluorescence signal shown in d. **f** Overlay of fluorescence signal from CD31-staining (red) in a ZR-75-1-based VTS and segmented PVs (transparent yellow) derived from this signal. **g** Segmented PVs (yellow) derived from CD31-fluorescence signal shown in d. **h** Segmentation of macrophages in a Sk-Br-3-based VTS. Pre-filtered fluorescence signal from CD11b-staining is shown in white, the resulting segmented and rendered macrophages are shown in solid yellow. Series of five frontal cuts in the xy-plane through VTS center, and at -100 µm, -50 µm, 50 µm and 100 µm from the center in the z-direction. Depth of each cut: 20 µm. **i** Segmentation of macrophages in a Sk-Br-3-based VTS. The pre-filtered fluorescence signal from CD11b-staining is shown in white, the resulting segmented and rendered macrophages are shown as red outlines in an overlay with the original fluorescence signal (white) and finally in solid yellow.

3D grid spacing: 50 µm, scale bars: 100 µm.

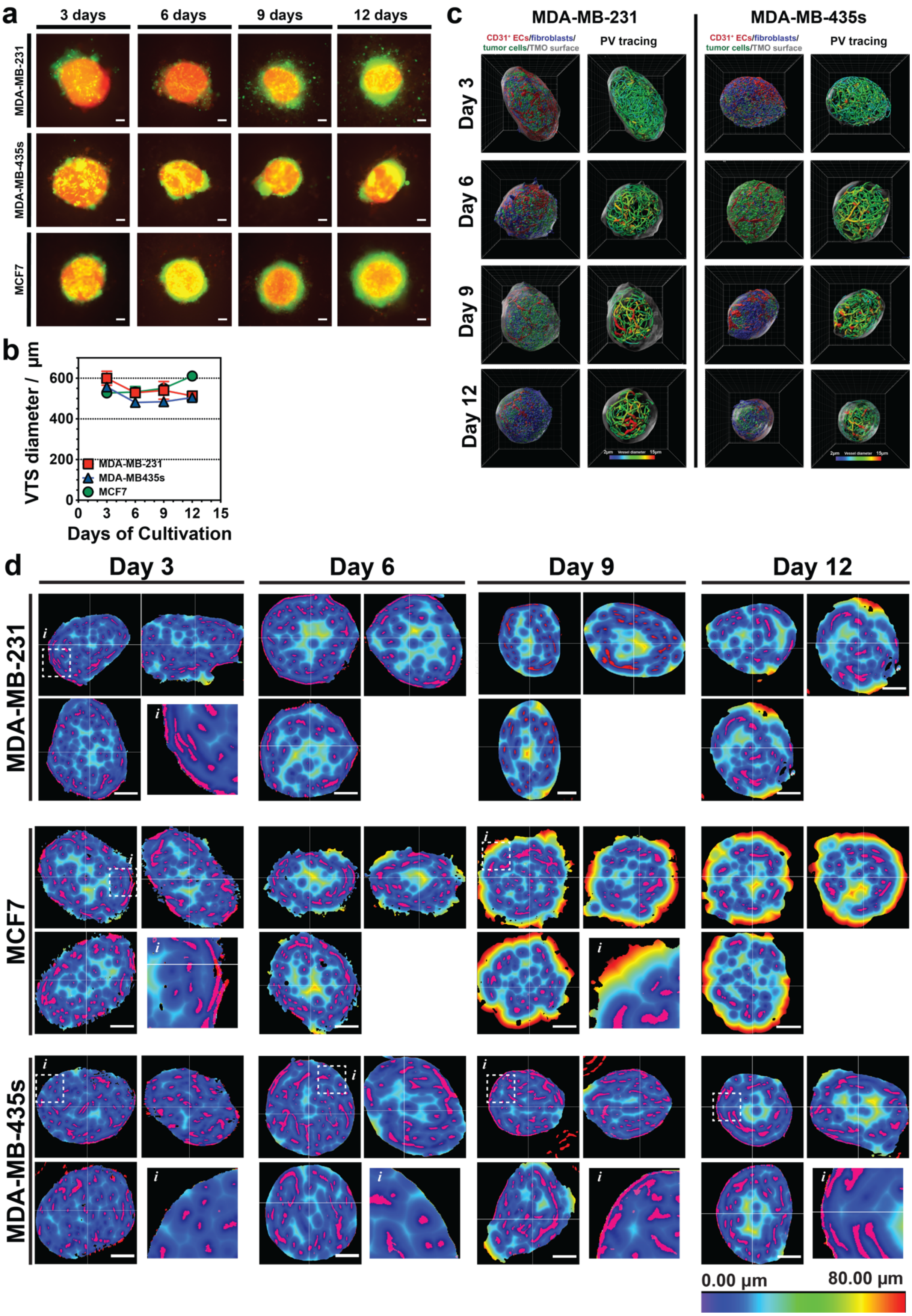

Supplementary Figure 2: Time course VTS maturation

a Epi-fluorescence images of VTSs during cultivation. VTSs were generated with three different tumor cell lines (green), THP-1, HUVEC, and NHDF (red) at a ratio of 1,000: 100: 2,000: 20,000. **b** Changes in VTS size over 12 days of cultivation. Volumes measured in BF-images images in viable VTSs. **c** Time-resolved changes of MDA-MB-231 and -435s-based VTSs over a cultivation period of 12 days. 3D-rendering of surfaces of the three imaged cellular compartments and 3D representation of traced PVs, colored according to segment mean diameter. **d** Heatmap displaying distances from the nearest PV (red) in VTSs generated from MDA-MB-231, -435s, or MCF7 tumor cells over a cultivation time of 12 days. In MCF7-VTSs – and to a lower extent MDA-MB-231-VTSs – PVs retread gradually from the VTS surface. In MDA-MB-231-VTSs a rudimentary PV network is formed first in the center of the VTS, which then extends to the surface after app. 9 days.

3D grid spacing: 50 µm, error bars: ± SEM, scale bars: 100 µm, n = 3 individual biological samples. Source data are provided as a Source Data file.

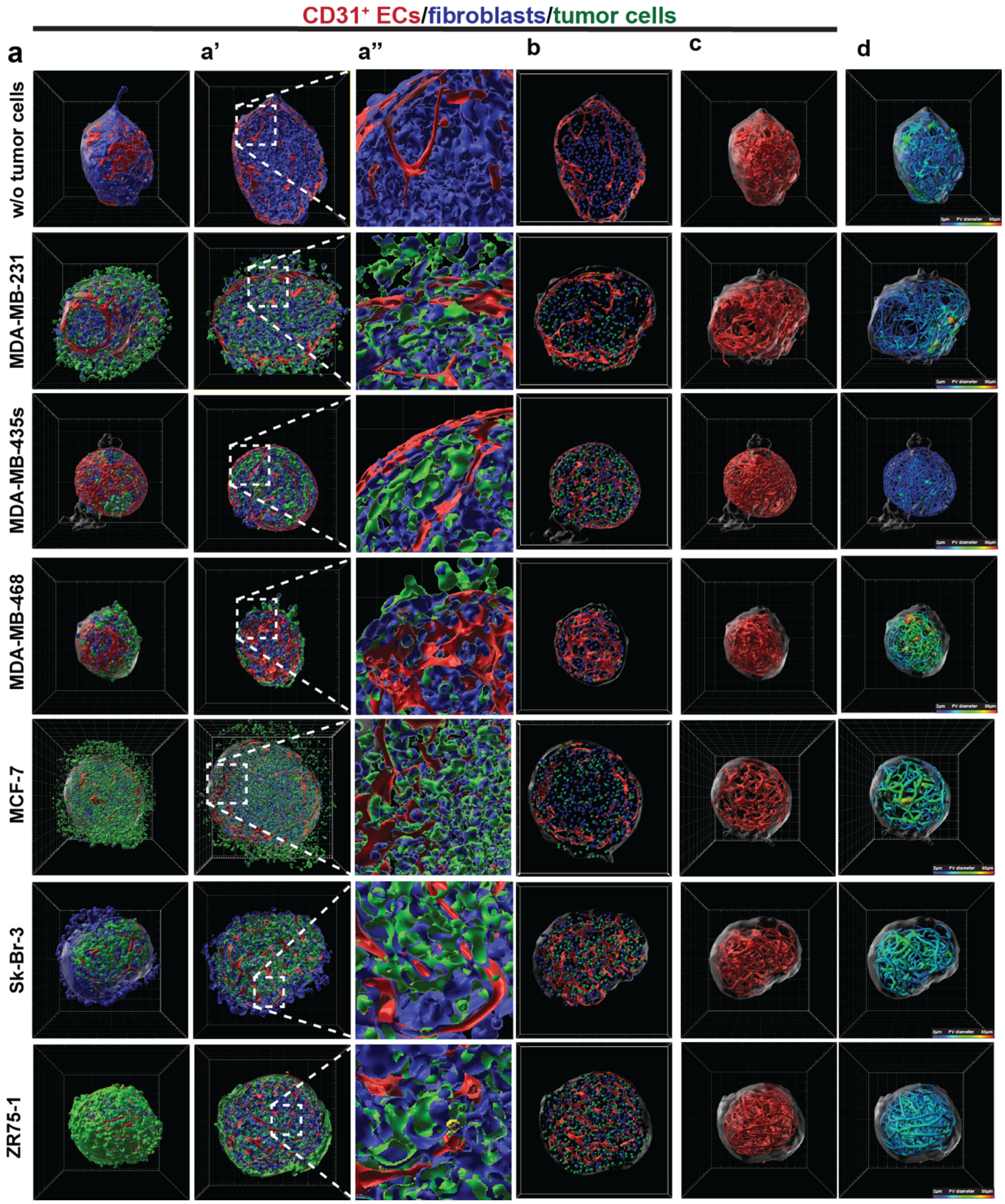

Supplementary Figure 3: Detailed evaluation of VTS structures

a 3D-rendering of surfaces of the three imaged cellular compartments (TCs (green), NHDF (blue), and $CD31^+$-ECs (red). VTS surface in transparent grey)) in VTSs generated with different tumor cell lines. View on the outer surface of VTS. **a'** frontal cut through respective VTSs, **a''** detailed view of a' displaying the spatial interaction of

TCs, fibroblasts, and ECs (view: 150 x 150 µm in the frontal plane). **b** Frontal cut through VTS center in the xy-plane, 50 µm depth in respective VTSs with PV ($CD31^{+}$-ECs (red), VTS surface in transparent grey) and TCs (green) and NHDF (blue) displayed as 10 µm orbs. **c** Surface rendering of PV (red) within VTS (transparent grey). **d** Traced PV in VTSs generated with different tumor cell lines. PV segments are color-coded according to mean diameter.

3D grid spacing: 50 µm.

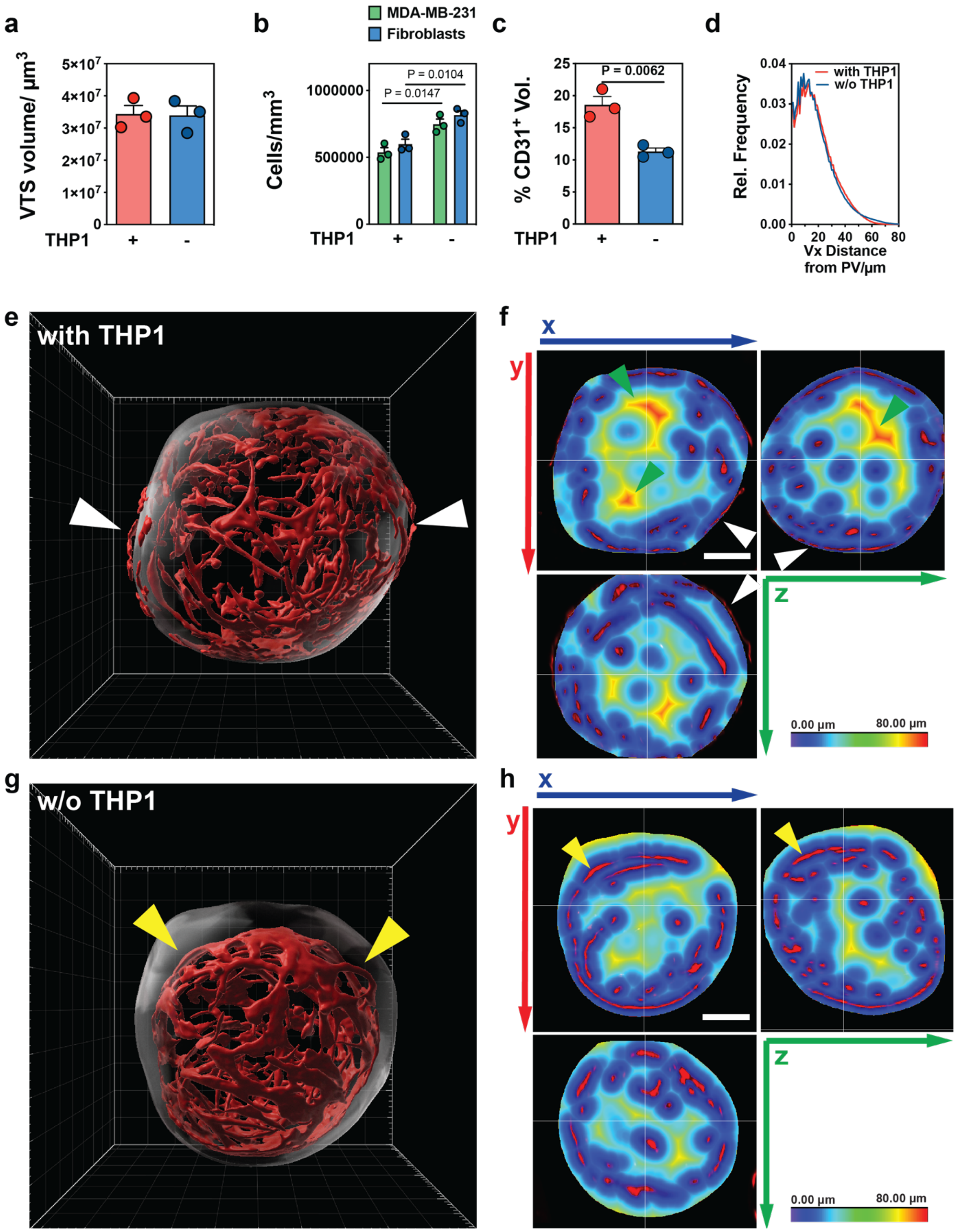

Supplementary Figure 4: Macrophage effect on VTS structure

a Volume of MDA-MB-231-based VTSs generated with or without the addition of THP-1 cells after 9 days of cultivation. **b** Density of tumor cells and fibroblasts in VTSs generated with or without the addition of THP-1 cells after 9 days of cultivation. **c**

Relative volume of CD31$^+$-PV-structures in VTSs generated with or without the addition of THP-1 cells after 9 days of cultivation. **d** Distribution of CD31$^+$-PV-structures in relationship to VTS volume in VTSs generated with or without the addition of THP-1 cells after 9 days of cultivation. Error bars: not shown for clarity. **e** Surface rendition of CD31$^+$-PV-structures in VTSs generated with the addition of THP-1 cells after 9 days of cultivation. PV-structures are formed on the surface of the VTS (white arrowheads). **f** Heatmap displaying distances from the nearest PV (red) in VTSs generated with the addition of THP-1 cells after 9 days of cultivation. PVs are located primarily close to and even on the surface (white arrowheads) of the VTS. Inside the VTS areas appear that are distal from the nearest PV (green arrowheads) **g** Surface rendition of CD31$^+$-PV-structures in VTSs generated without the addition of THP-1 cells after 9 days of cultivation. PV-structures do not reach the surface of the VTS (yellow arrowheads). **h** Heatmap displaying distances from the nearest PV (red) in VTSs generated with the addition of THP-1 cells after 9 days of cultivation. PVs are located inside the VTS at some distance to the surface (yellow arrowheads).

3D grid spacing: 50 µm, scale bars: 100 µm, error bars: ± SEM, analyzed with unpaired two-tailed *t*-test, n = 3 individual biological samples. Source data are provided as a Source Data file.

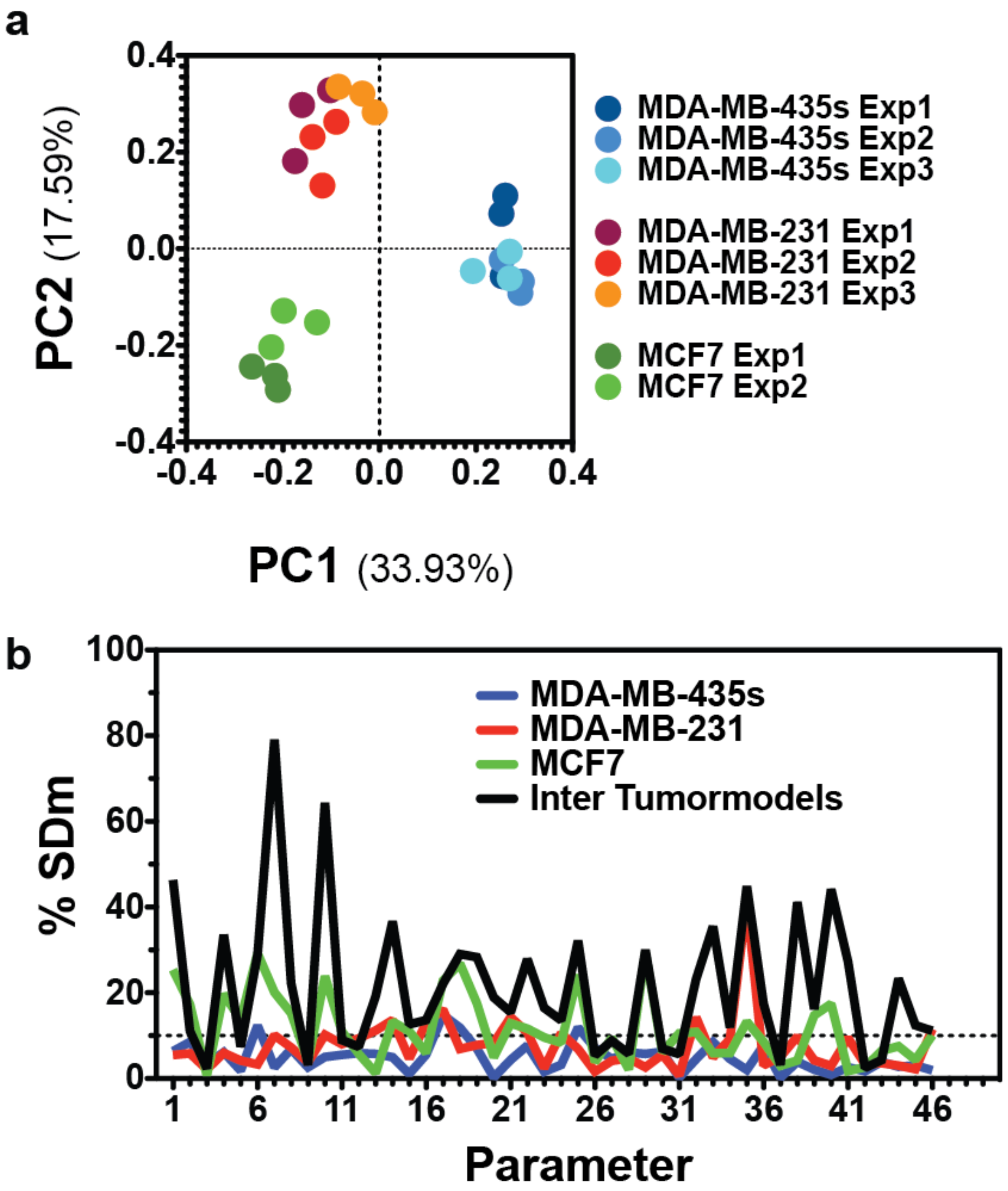

Supplementary Figure 5: Assay reliability

a Results from PCA on 2 (MCF7) and 3 (MDA-MB-231, -435s) descriptive datasets obtained in independent experiments for the respective control group. The results from compartmental, tracing, distance transformation, and cellular localization analysis were compiled for each of the 3 replicates in each experiment, and the resulting dataset was subjected to PCA. Results from individual cell lines clustered together. **b** Standard deviation of the mean (SDm) obtained from independent experiments with the same tumor cell line (MCF7, MDA-MB-231, or -435s) listed as a percentage of the

mean for each calculated parameter from compartmental, tracing, distance transformation, and cellular localization analysis. The percental SDm for the respective average values with the three different cell lines is given for comparison. The data shows that even over several independent experiments, divergence within a tumor model is significantly lower than between different models.

N = 3 individual biological samples; error bars not shown for clarity. Exp: experiment. Source data are provided as a Source Data file.

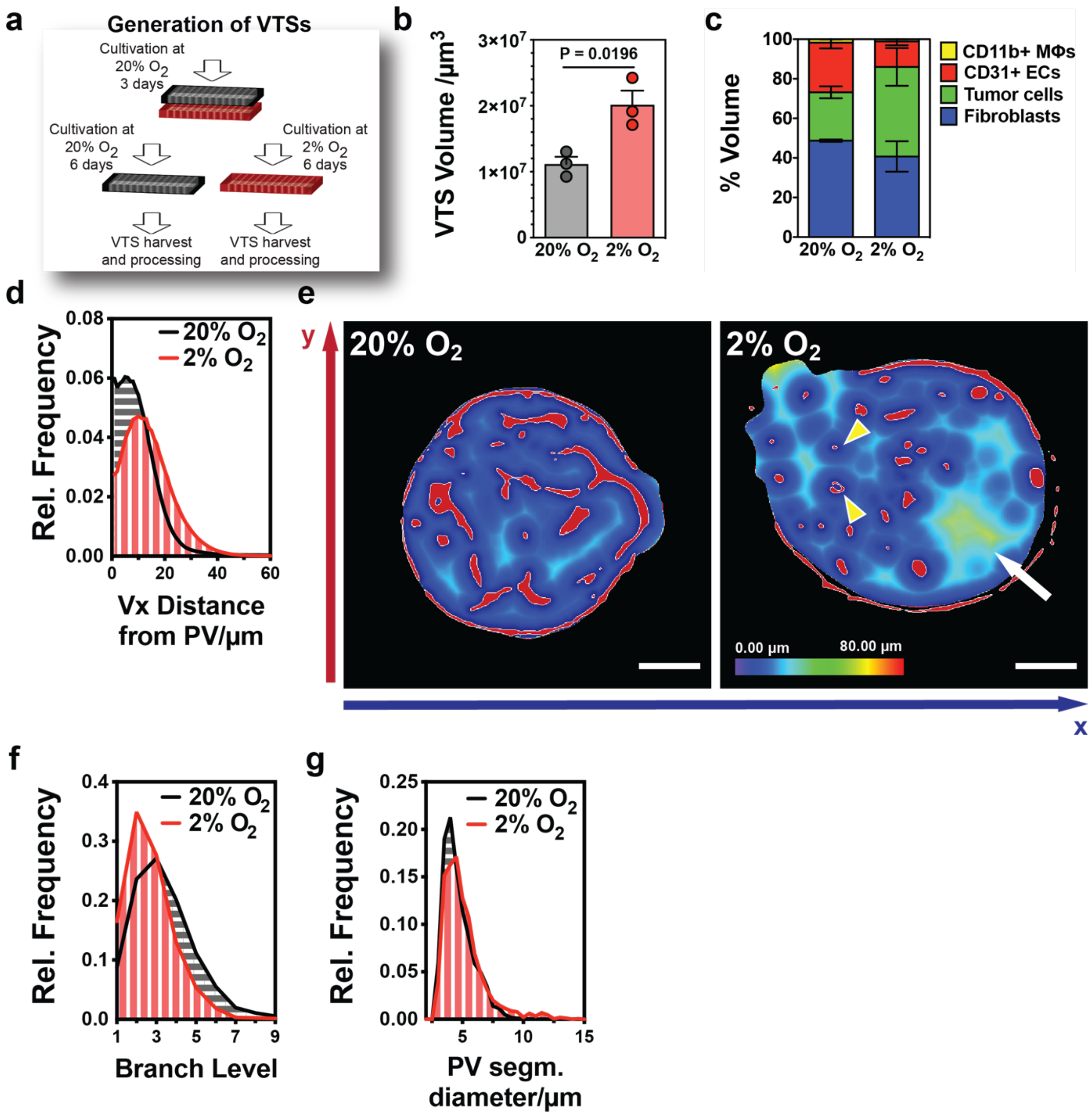

Supplementary Figure 6: Effect of hypoxia on PV development

a Schematic overview of the procedure for cultivation of VTSs under hypoxia (2% O_2). **b** Volume of MDA-MB-435s based VTSs cultivated under normoxia (20% O_2) or hypoxia (2% O_2) after 9 days of cultivation. **c** Relative volume of cellular compartments in VTSs cultivated under normoxia (20% O_2) or hypoxia (2% O_2). **d** Distribution of voxel distances within VTS from nearest PV in VTSs cultivated under normoxia or hypoxia, indicating larger areas poorly supplied by PVs under hypoxia. Error bars not shown for clarity. **e** 2D Heatmap displaying distances from the nearest PV (red) cultivated under normoxia or hypoxia. Under hypoxia, PVs are still abundant on the VTS surface, but

the PV network inside the VTS is largely dissolved and fragmented (yellow arrowheads). In the center, some areas are poorly interspersed with PVs (white arrow). **f** Distribution of PV-segment branch levels in VTSs cultivated under normoxia or hypoxia showing a reduced complexity of the PV networks after cultivation under hypoxia. Error bars not shown for clarity. **g** Distribution of PV-segment mean diameters in VTSs cultivated under normoxia or hypoxia showing a slightly increased size of PVs after cultivation under hypoxia. Error bars: not shown for clarity.

Scale bars: 100 µm, error bars: ± SEM, analyzed with unpaired two-tailed *t*-test, n = 3 individual biological samples. Source data are provided as a Source Data file**.**

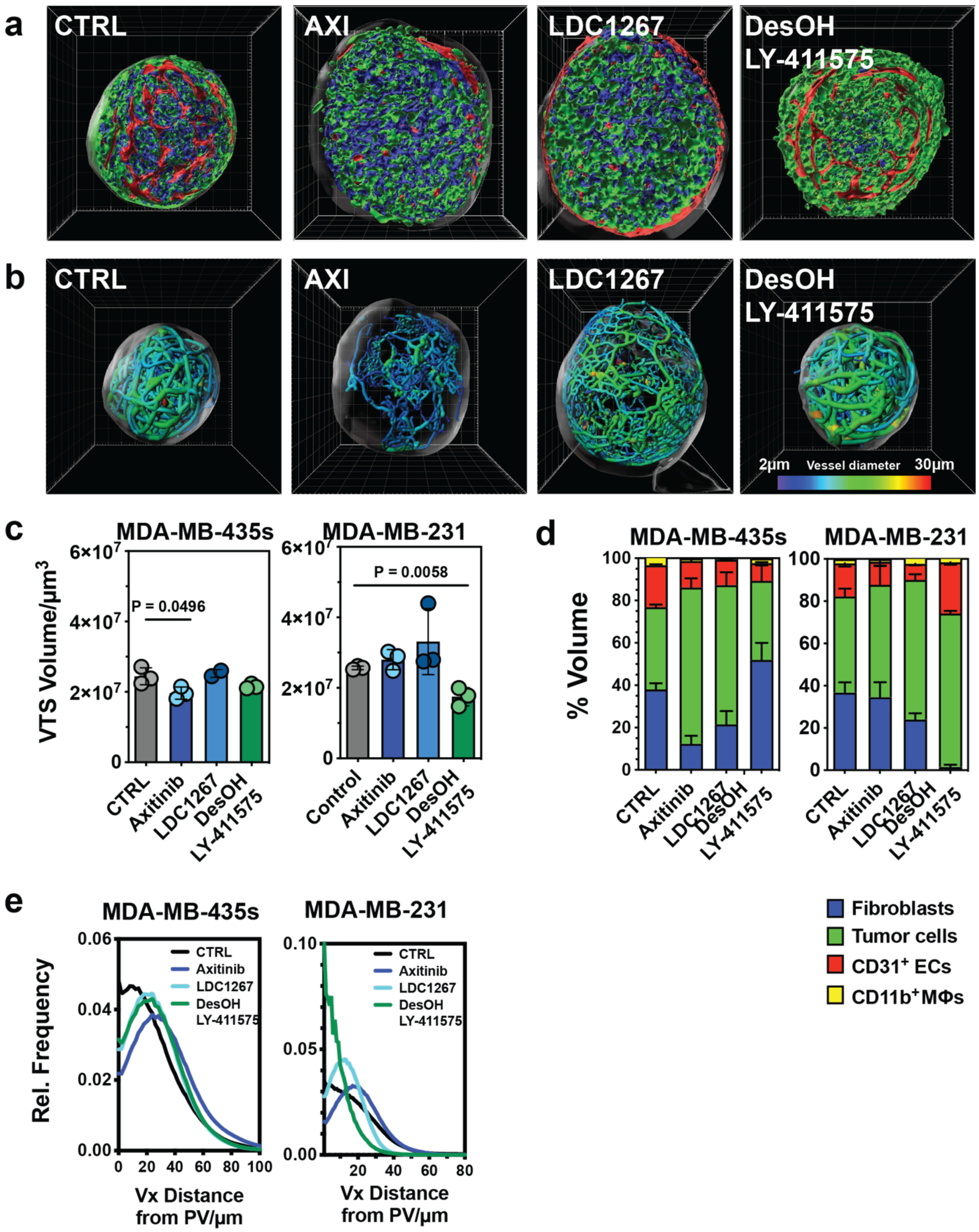

Supplementary Figure 7: Angiogenesis inhibitors in MDA-MB-231-VTSs

a Architecture of MDA-MB-231-based VTSs after treatment with AXI, LDC 1267, or DesOH LY-411575. 3D-rendering of $CD31^+$-PVs (red), TCs (green), and fibroblasts (blue). Frontal cut through VTS center in the xy-plane. **b** Tracing of PV networks in MDA-MB-231-based VTSs treated with AXI, LDC 1267, or DesOH LY-411575. 3D

rendering of surfaces and segments colored according to diameter. **c** Volumes of MDA-MB-435s or 231-based VTSs treated with AXI, LDC 1267, or DesOH LY-411575. **d** Relative volume of cellular compartments in MDA-MB-435s or 231-based VTSs treated with AXI, LDC 1267, or DesOH LY-411575. **e** Distribution of voxel distances within VTS from nearest PV in MDA-MB-435s or 231-based VTSs treated with AXI, LDC 1267, or DesOH LY-411575. N = 3, error bars: not shown for clarity.

3D grid spacing: 50 µm, error bars: ± SEM, analyzed with unpaired two-tailed *t*-test, n = 3 individual biological samples. Source data are provided as a Source Data file.

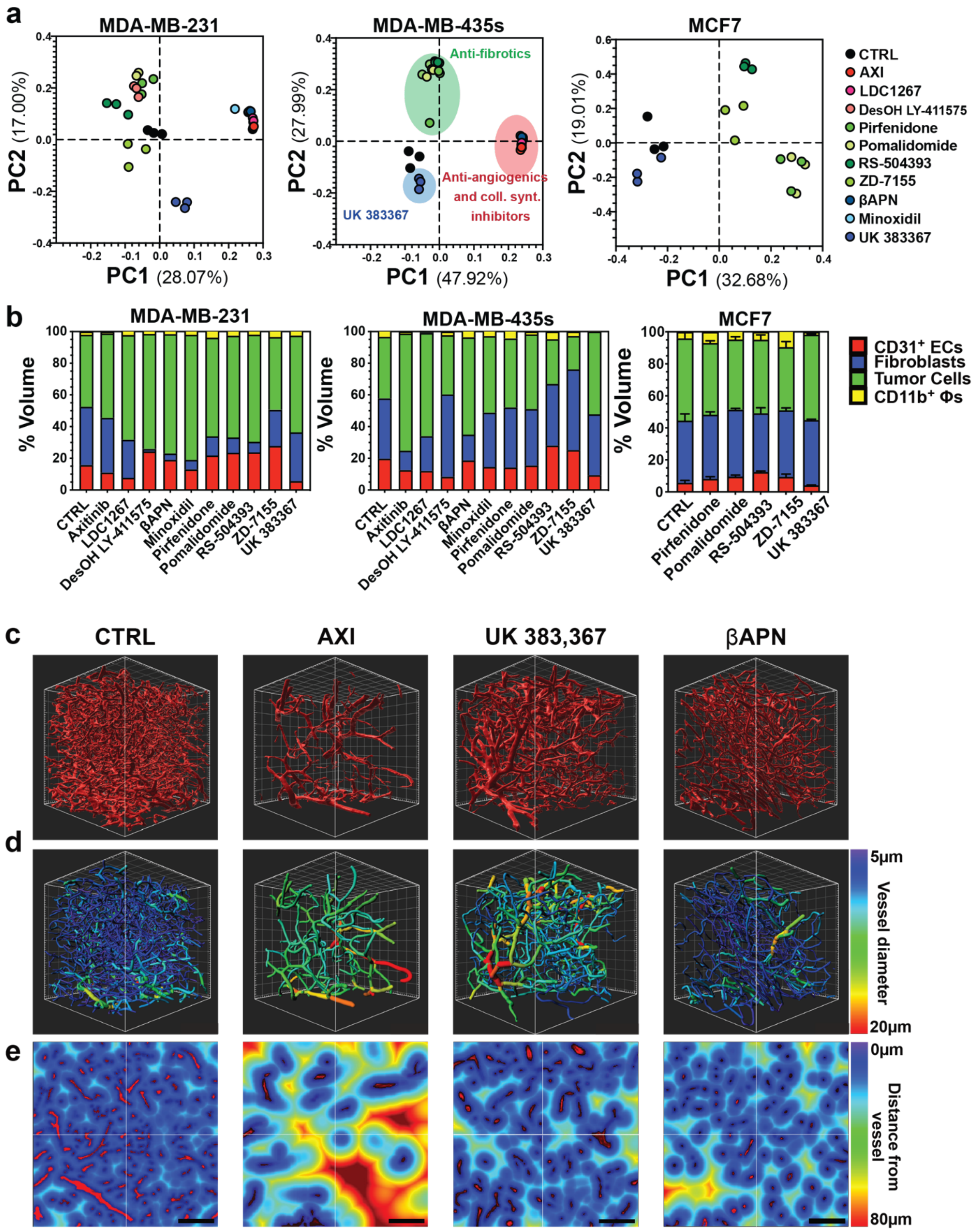
a
MDA-MB-231
MDA-MB-435s
MCF7
PC1 (28.07%)
PC2 (17.00%)
PC1 (47.92%)
PC2 (27.99%)
PC1 (32.68%)
PC2 (19.01%)
Anti-fibrotics
Anti-angiogenics and coll. synt. inhibitors
UK 383367
CTRL
AXI
LDC1267
DesOH LY-411575
Pirfenidone
Pomalidomide
RS-504393
ZD-7155
βAPN
Minoxidil
UK 383367
b
% Volume
CD31⁺ ECs
Fibroblasts
Tumor Cells
CD11b⁺ Φs
CTRL
Axitinib
LDC1267
DesOH LY-411575
βAPN
Minoxidil
Pirfenidone
Pomalidomide
RS-504393
ZD-7155
UK 383367
c
CTRL
AXI
UK 383,367
βAPN
d
5μm
Vessel diameter
20μm
e
0μm
Distance from vessel
80μm

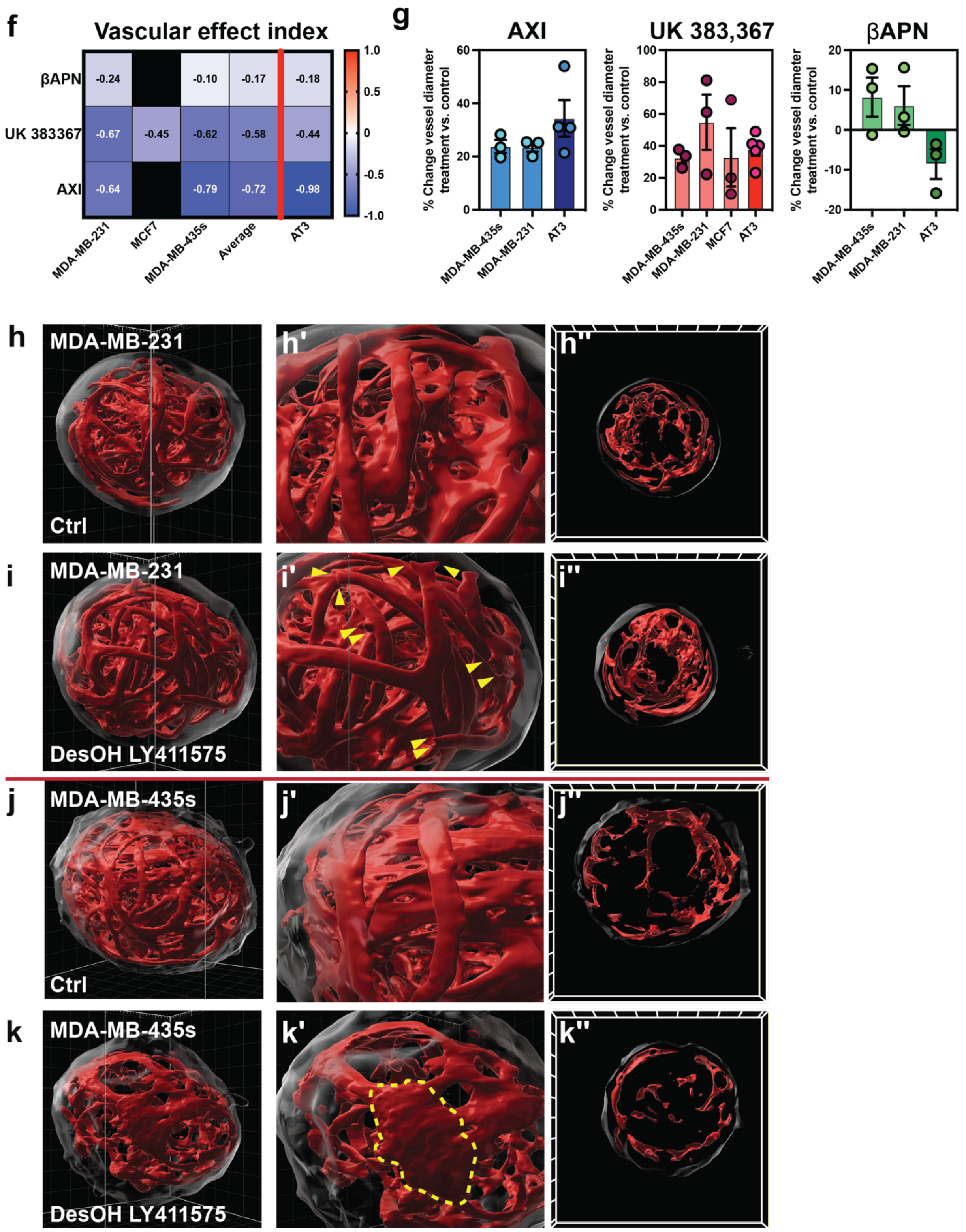

Supplementary Figure 8: Effect of DesOH LY411575

a Results from PCA on descriptive datasets after treatment with various agents in MDA-MB-231-, MDA-MB-435s-, and MCF7-based VTSs. **b** Relative volume of cellular compartments in MDA-MB-231-, MDA-MB-435s-, and MCF7-based VTSs after

treatment with various agents. **c** Network of perfused vessels in murine AT3 breast carcinomas after treatment with AXI, UK 383367, or βAPN. All treatments reduced perfused vessel volume, AXI was the most effective drug in that respect Intravital staining with an Alexa647-labelled CD105 antibody. Shown are rendered surfaces after reconstruction. 3D grid width: 50 µm. **d** Tracing of perfused vessels in murine AT3 breast carcinomas after treatment with AXI, UK 383367, or βAPN. 3D rendering of surfaces and segments colored according to diameter. 3D grid width: 50 µm. **e** 2D Heatmap displaying distances from the nearest perfused vessel (red) in murine AT3 breast carcinomas after treatment with AXI, UK 383367, or βAPN. In treated tumors, perfused vessel density is significantly reduced. Consequently, large portions of the tumor are located at increased distances from the nearest vessel and under higher risk of hypoxia. **f** Vascular effect index (VEI) was calculated after vessel parametrization in murine AT3 breast carcinomas after treatment with AXI, UK 383367, or βAPN. The VEI was calculated using the same methods and algorithms as in VTSs. For comparison VEIs in VTSs after treatment with the same agents are shown (data from MDA-MB-435s, MDA-MB-231 and MCF7 VTSs, and the average VEI observed in the VTSs). Effects in the murine model generally align with the results from the VTS experiments. **g** Changes in average vessel diameter in MDA-MB-435s, MDA-MB-231 and MCF7 (UK 383367 only) VTSs and in murine AT3 breast carcinomas after treatment with AXI, UK 383367, or βAPN. n = 3 individual biological samples (VTS treatment); n = 4 individual tumors (AT3 AXI treatment); n = 5 individual tumors (AT3 UK 383367 treatment), and n = 3 individual tumors (AT3 βAPN treatment). **h** Surface rendering of $CD31^+$-PV-structures in MDA-MB-231-based VTSs. Full view of VTS, detail (**h'**), and (**h''**) 50 µm thick frontal cut through the center. **i** Surface rendering of $CD31^+$-PV-structures in MDA-MB-231-based VTSs after

treatment with DesOH LY411575. Full view of VTS, detail (**i'**), and (**i''**) 50 µm thick frontal cut through the center. The PV network is denser, reaching deeper into the center. On several segments, spore-like protrusions, indicating tip cells are visible (yellow arrowheads). **j** Surface rendering of CD31^{+}-PV-structures in MDA-MB-435s-based VTSs. Full view of VTS, detail (**j'**), and (**j''**) 50 µm thick frontal cut through the center. **k** Surface rendering of CD31^{+}-PV-structures in MDA-MB-435s-based VTSs after treatment with DesOH LY411575. Full view of VTS, detail (**k'**), and (**k''**) 50 µm thick frontal cut through the center. The PV network is reduced in density. Fewer structures reach into the center. Segments appear to be fused into plate-like structures (yellow outline).

3D grid spacing: 50 µm, error bars: ± SEM, n = 3 individual biological samples if not otherwise indicated. Source data are provided as a Source Data file.

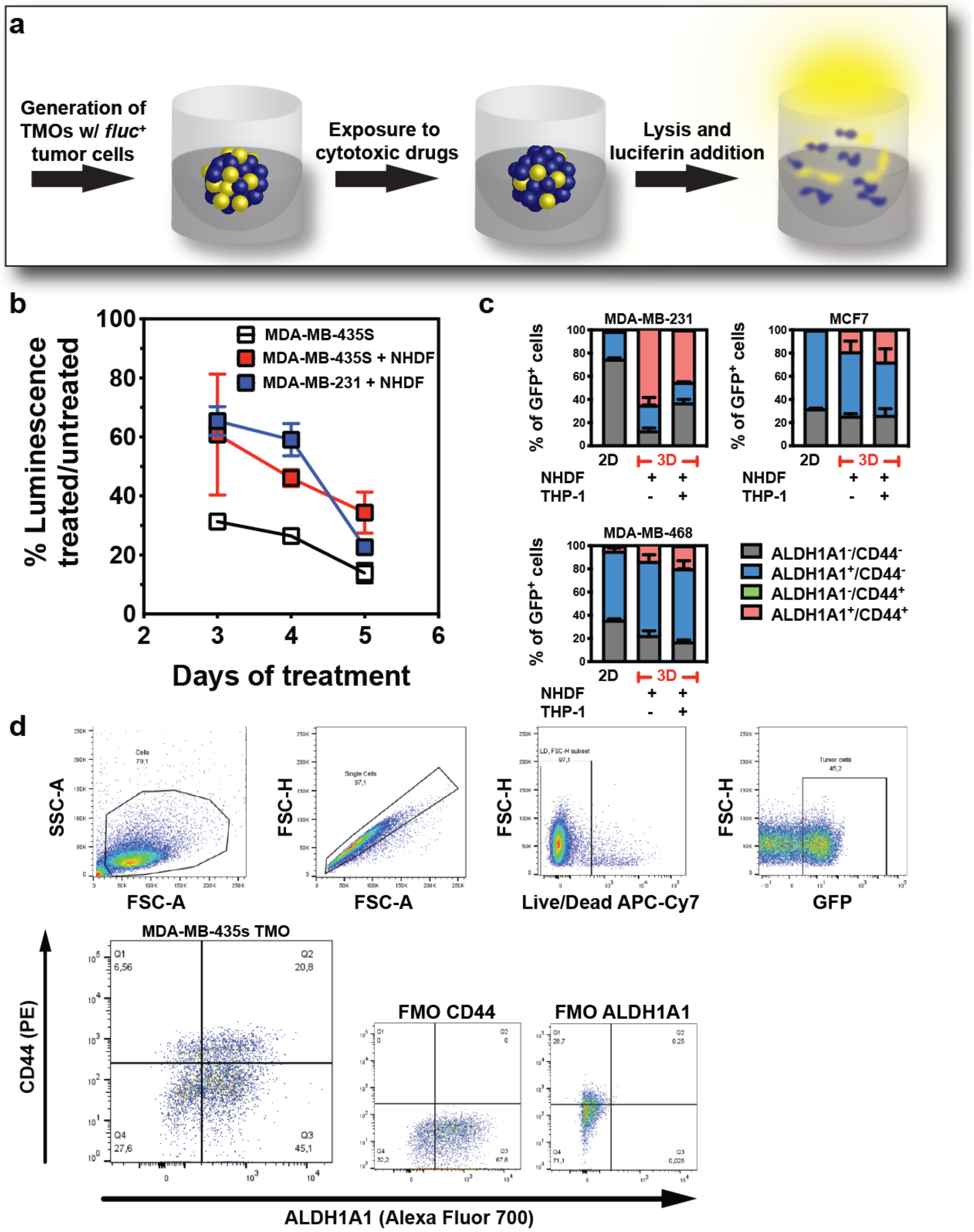

Supplementary Figure 9: Cell toxicity assay

a Schematic representation of cytotoxic evaluation in complex VTSs: TCs were engineered by lentiviral infection to stably express firefly luciferase (*fluc*). Generation of VTSs with these TCs allowed for quantification of surviving TCs after exposure to cytotoxic drugs *via* measurement of luciferase activity. In a single step, VTSs were

lysed and exposed to luciferin to initiate luminescence. **b** Remaining signal in different VTSs and MCTS exposed to 1 μM PTX, for 3, 4, and 5 days. **c** Results from FACS analysis of ALDH1A and CD44 expression in TCs cultivated either in 2D or in 3D as VTSs in various cellular setups n = 2 individual samples. **d** FACS gating strategy to quantify ALDH1A and CD44 expression in TCs. Exemplary display of FACS analysis of ALDH1A and CD44 expression in MDA-MB-435s cultivated in 3D as VTSs in various cellular setups.

Error bars: ± SEM. Source data are provided as a Source Data file.

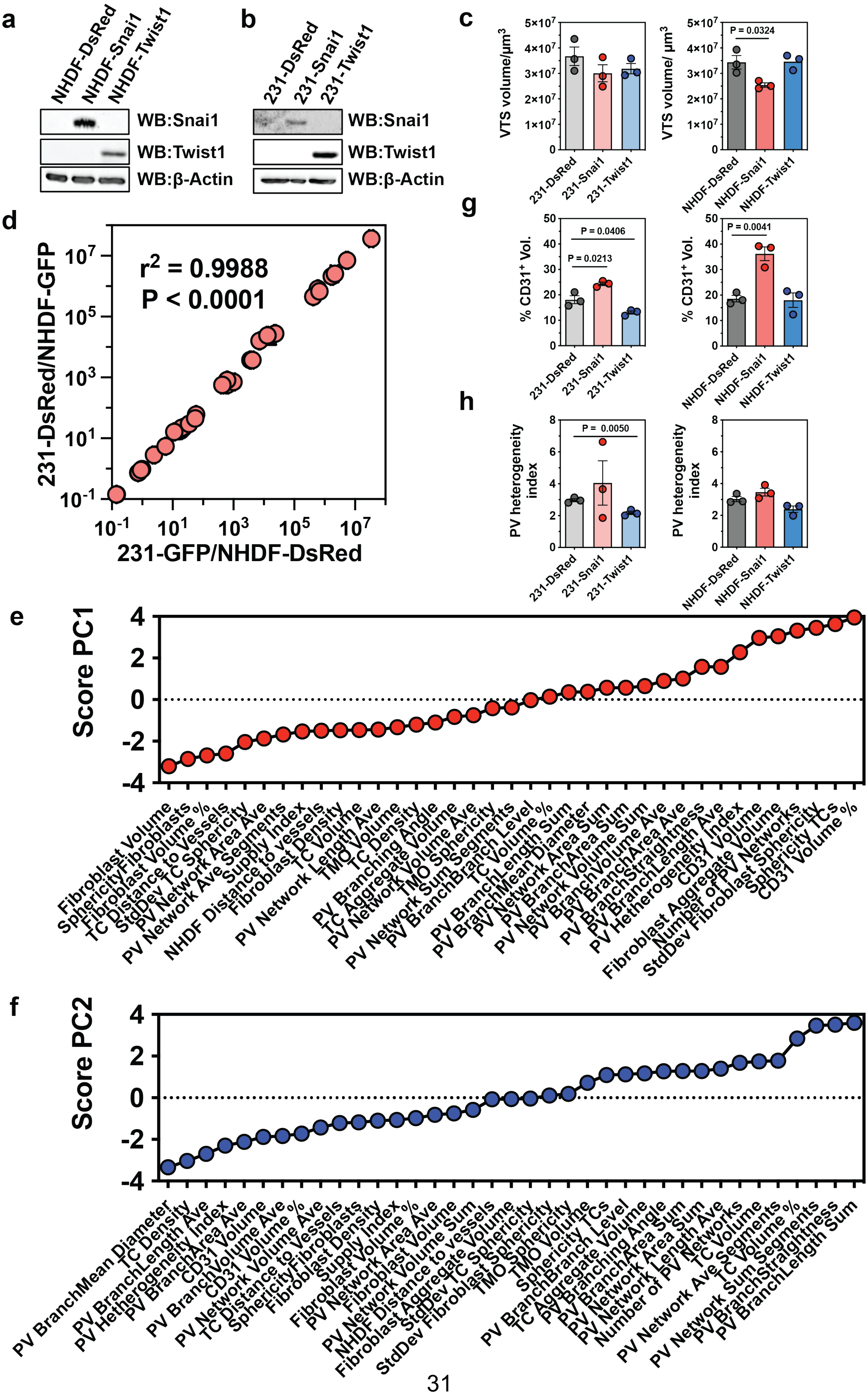
a
NHDF-DsRed
NHDF-Snai1
NHDF-Twist1
WB:Snai1
WB:Twist1
WB:β-Actin
b
231-DsRed
231-Snai1
231-Twist1
WB:Snai1
WB:Twist1
WB:β-Actin
c
VTS volume/μm³
VTS volume/ μm³
5×10⁷
4×10⁷
3×10⁷
2×10⁷
1×10⁷
0
P = 0.0324
231-DsRed
231-Snai1
231-Twist1
NHDF-DsRed
NHDF-Snai1
NHDF-Twist1
d
r² = 0.9988
P < 0.0001
231-DsRed/NHDF-GFP
231-GFP/NHDF-DsRed
10⁻¹
10¹
10³
10⁵
10⁷
g
% CD31⁺ Vol.
P = 0.0406
P = 0.0213
P = 0.0041
50
40
30
20
10
0
h
PV heterogeneity index
P = 0.0050
8
6
4
2
0
e
Score PC1
4
2
0
-2
-4
Fibroblast Volume
SphericityFibroblasts
Fibroblast Volume %
TC Distance to Vessels
StdDev TC Sphericity
PV Network Area Ave
PV Network Ave Segments
Supply Index
NHDF Distance to vessels
Fibroblast Density
TC Volume
PV Network Length Ave
TMO Volume
TC Density
PV Branching Angle
TC Aggregate Volume
PV Network Volume Ave
TMO Sphericity
PV Network Sum Segments
PV BranchBranch Level
TC Volume %
PV BranchLength Sum
PV BranchMean Diameter
PV Network Area Sum
PV BranchArea Sum
PV Network Volume Sum
PV BranchVolume Ave
PV BranchArea Ave
PV BranchStraightness
PV BranchLength Ave
PV Hetherogeneity Index
CD31 Volume
Fibroblast Aggregate Volume
Number of PV Networks
StdDev Fibroblast Sphericity
Sphericity TCs
CD31 Volume %
f
Score PC2
4
2
0
-2
-4
PV BranchMean Diameter
TC Density
PV BranchLength Ave
PV Hetherogeneity Index
PV BranchArea Ave
CD31 Volume
PV BranchVolume Ave
CD31 Volume %
PV Network Volume Ave
TC Distance to Vessels
SphericityFibroblasts
Fibroblast Density
Supply Index
Fibroblast Volume %
PV Network Area Ave
Fibroblast Volume
PV Network Volume Sum
NHDF Distance to vessels
Fibroblast Aggregate Volume
StdDev TC Sphericity
StdDev Fibroblast Sphericity
TMO Sphericity
TMO Volume
Sphericity TCs
PV BranchBranch Level
TC Aggregate Volume
PV Branching Angle
PV BranchArea Sum
PV Network Area Sum
PV Network Length Ave
Number of PV Networks
TC Volume
PV Network Ave Segments
TC Volume %
PV Network Sum Segments
PV BranchStraightness
PV BranchLength Sum

Supplementary Figure 10: Genetic Manipulations

a Western blot for Twist1 and Snai1 of NHDF lysates after lentiviral transfection to stably overexpress one of the transcription factors. **b** Western blot for Twist1 and Snai1 of MDA-MB-231 lysates after lentiviral transfection to stably overexpress one of the transcription factors. Uncropped images of the probed membranes are provided in the Source data file for this figure. **c** Volume of VTSs generated with MDA-MB-231 or NHDF stably overexpressing Snai1 or Twist1 after 9 days of cultivation. **d** Correlation between means of descriptive parameters obtained for VTSs generated from MDA-MB-231-DsRed cells and NHDF-GFP (+ HUVEC, + THP-1) vs. MDA-MB-231-GFP cells and NHDF-DsRed (+ HUVEC, + THP-1). Parameters were obtained from compartmental, tracing, distance transformation, and cellular localization analysis. Results from two-tailed correlation analysis with C.I. = 95%, and calculated Pearson correlation coefficient. **e** Score sheet for PC1 from principal component analysis of effects from Snai1 or Twist1 OE in MDA-MB-231 tumor cells or NHDFs. **f** Score sheet for PC2 from principal component analysis of effects from Snai1 or Twist1 OE in MDA-MB-231 tumor cells or NHDFs. **g** Relative $CD31^+$-PV volumes in VTSs after Snai1 or Twist1 OE in MDA-MB-231 tumor cells or NHDFs. **h** PV heterogeneity index in VTSs after Snai1 or Twist1 OE in MDA-MB-231 tumor cells or NHDFs.

Error bars: ± SEM, analyzed with unpaired two-tailed *t*-test, n = 3 individual biological samples. Source data are provided as a Source Data file.

a

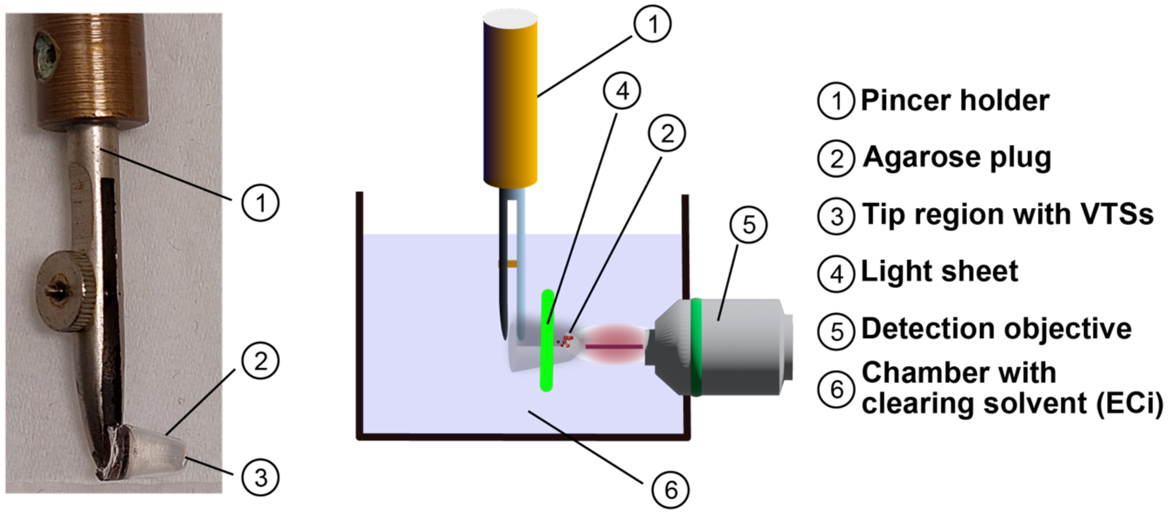

b

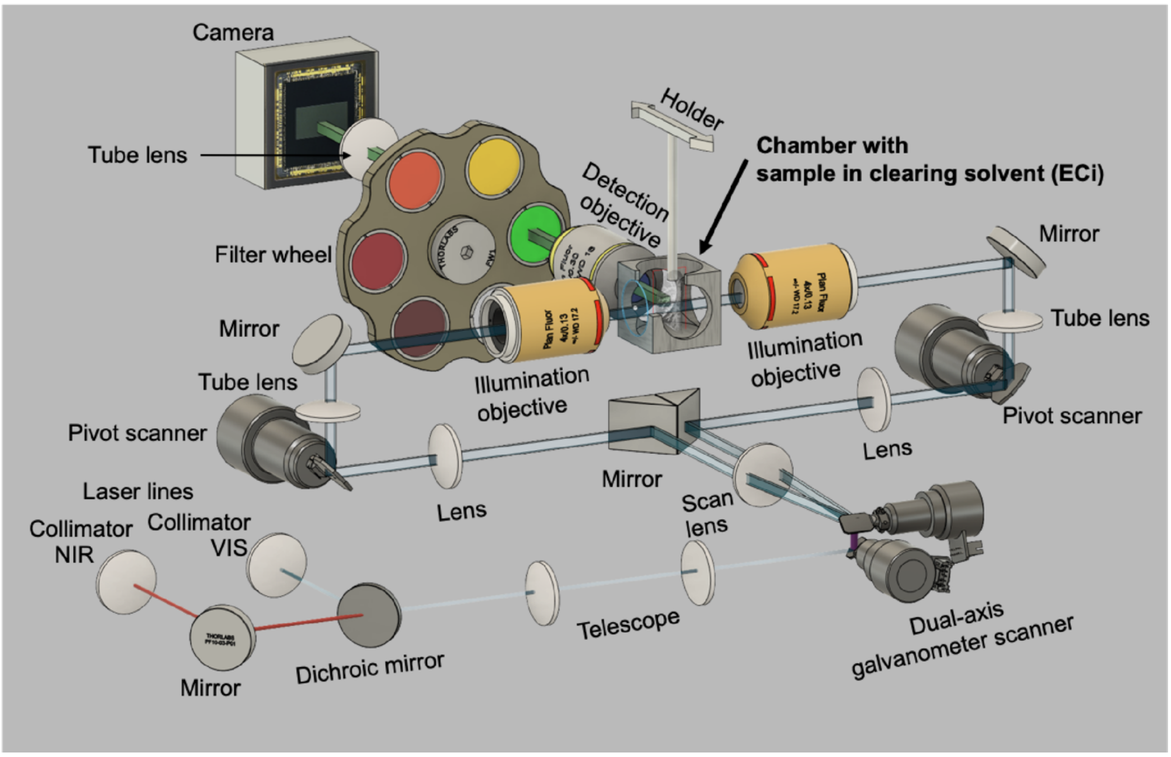

Supplementary Figure 11: Setup of the light sheet fluorescence microscope

a Attachment of the conical agarose plug that contains the VTSs at its tip to a pincer holder (left) and schematic representation of the attached sample submerged in the imaging chamber of the LSFM (right). **b** Schematic representation of the light sheet fluorescence microscope used for the experiments. Specifications for the individual parts are given in the materials and methods section.

Supplementary References

1. Subik, K. et al. The Expression Patterns of ER, PR, HER2, CK5/6, EGFR, Ki-67 an,d AR by Immunohistochemical Analysis in Breast Cancer Cell Lines. *Breast Cancer (Auckl)* **4**, 35-41 (2010).
2. Cheang, M.C. et al. Basal-like breast cancer defined by five biomarkers has superior prognostic value than triple-negative phenotype. *Clin Cancer Res* **14**, 1368-1376 (2008).
3. Korch, C. et al. Authentication of M14 melanoma cell line proves misidentification of MDA-MB-435 breast cancer cell line. *Int J Cancer* **142**, 561-572 (2018).
4. Hu-Lowe, D.D. et al. Nonclinical antiangiogenesis and antitumor activities of axitinib (AG-013736), an oral, potent, and selective inhibitor of vascular endothelial growth factor receptor tyrosine kinases 1, 2, 3. *Clin Cancer Res* **14**, 7272-7283 (2008).
5. Mancuso, M.R. et al. Rapid vascular regrowth in tumors after reversal of VEGF inhibition. *J Clin Invest* **116**, 2610-2621 (2006).
6. Curry, C.L. et al. Gamma secretase inhibitor blocks Notch activation and induces apoptosis in Kaposi's sarcoma tumor cells. *Oncogene* **24**, 6333-6344 (2005).
7. Paolino, M. et al. The E3 ligase Cbl-b and TAM receptors regulate cancer metastasis via natural killer cells. *Nature* **507**, 508-512 (2014).
8. Mu, X.Y. et al. RS 504393 inhibits M-MDSCs recruiting in immune microenvironment of bladder cancer after gemcitabine treatment. *Mol Immunol* **109**, 140-148 (2019).
9. Furuichi, K. et al. CCR2 signaling contributes to ischemia-reperfusion injury in kidney. *J Am Soc Nephrol* **14**, 2503-2515 (2003).
10. D'Amato, R.J., Lentzsch, S. & Rogers, M.S. Pomalidomide is strongly antiangiogenic and teratogenic in relevant animal models. *Proc Natl Acad Sci U S A* **110**, E4818 (2013).
11. Weingartner, S. et al. Pomalidomide is effective for prevention and treatment of experimental skin fibrosis. *Ann Rheum Dis* **71**, 1895-1899 (2012).
12. Mediavilla-Varela, M., Boateng, K., Noyes, D. & Antonia, S.J. The anti-fibrotic agent pirfenidone synergizes with cisplatin in killing tumor cells and cancer-associated fibroblasts. *BMC Cancer* **16**, 176 (2016).
13. Kozono, S. et al. Pirfenidone inhibits pancreatic cancer desmoplasia by regulating stellate cells. *Cancer Res* **73**, 2345-2356 (2013).
14. Tang, H.T. et al. Angiotensin II induces type I collagen gene expression in human dermal fibroblasts through an AP-1/TGF-beta1-dependent pathway. *Biochemical and biophysical research communications* **385**, 418-423 (2009).
15. Zhang, H. et al. Structure of the Angiotensin receptor revealed by serial femtosecond crystallography. *Cell* **161**, 833-844 (2015).
16. Bai, M. et al. BMP1 inhibitor UK383,367 attenuates renal fibrosis and inflammation in CKD. *Am J Physiol Renal Physiol* **317**, F1430-F1438 (2019).
17. Strom, T.B., Bjune, K. & Leren, T.P. Bone morphogenetic protein 1 cleaves the linker region between ligand-binding repeats 4 and 5 of the LDL receptor and makes the LDL receptor non-functional. *Hum Mol Genet* **29**, 1229-1238 (2020).
18. Zuurmond, A.M., van der Slot-Verhoeven, A.J., van Dura, E.A., De Groot, J. & Bank, R.A. Minoxidil exerts different inhibitory effects on gene expression of

lysyl hydroxylase 1, 2, and 3: implications for collagen cross-linking and treatment of fibrosis. *Matrix Biol* **24**, 261-270 (2005).
19. Eisinger-Mathason, T.S. et al. Hypoxia-dependent modification of collagen networks promotes sarcoma metastasis. *Cancer discovery* **3**, 1190-1205 (2013).
20. Murad, S. & Pinnell, S.R. Suppression of fibroblast proliferation and lysyl hydroxylase activity by minoxidil. *J Biol Chem* **262**, 11973-11978 (1987).
21. Tang, S.S., Trackman, P.C. & Kagan, H.M. Reaction of aortic lysyl oxidase with beta-aminopropionitrile. *J Biol Chem* **258**, 4331-4338 (1983).
22. Erler, J.T. et al. Lysyl oxidase is essential for hypoxia-induced metastasis. *Nature* **440**, 1222-1226 (2006).
23. Rossow, L. et al. LOX-catalyzed collagen stabilization is a proximal cause for intrinsic resistance to chemotherapy. *Oncogene* **37**, 4921-4940 (2018).
