# Supplementary material for: A vascularized breast cancer spheroid platform for the ranked evaluation of tumor microenvironment-targeted drugs by light sheet fluorescence microscopy
Source: Nat Commun. 2024 Apr 27;15:3599. doi: 10.1038/s41467-024-48010-z (PMC11055956; doi:10.1038/s41467-024-48010-z)
Supplement: Supplementary file 2 — Reporting Summary [file 41467_2024_48010_MOESM2_ESM.pdf]

nature portfolio

Corresponding author(s): Erik Henke

Last updated by author(s): Mar 26, 2024

Reporting Summary

Nature Portfolio wishes to improve the reproducibility of the work that we publish. This form provides structure for consistency and transparency in reporting. For further information on Nature Portfolio policies, see our Editorial Policies and the Editorial Policy Checklist.

Statistics

For all statistical analyses, confirm that the following items are present in the figure legend, table legend, main text, or Methods section.

n/a	Confirmed	
☐	☒	The exact sample size (*n*) for each experimental group/condition, given as a discrete number and unit of measurement
☐	☒	A statement on whether measurements were taken from distinct samples or whether the same sample was measured repeatedly
☐	☒	The statistical test(s) used AND whether they are one- or two-sided<br>*Only common tests should be described solely by name; describe more complex techniques in the Methods section.*
☒	☐	A description of all covariates tested
☐	☒	A description of any assumptions or corrections, such as tests of normality and adjustment for multiple comparisons
☐	☒	A full description of the statistical parameters including central tendency (e.g. means) or other basic estimates (e.g. regression coefficient) AND variation (e.g. standard deviation) or associated estimates of uncertainty (e.g. confidence intervals)
☐	☒	For null hypothesis testing, the test statistic (e.g. *F*, *t*, *r*) with confidence intervals, effect sizes, degrees of freedom and *P* value noted<br>*Give P values as exact values whenever suitable.*
☒	☐	For Bayesian analysis, information on the choice of priors and Markov chain Monte Carlo settings
☒	☐	For hierarchical and complex designs, identification of the appropriate level for tests and full reporting of outcomes
☒	☐	Estimates of effect sizes (e.g. Cohen's *d*, Pearson's *r*), indicating how they were calculated

Our web collection on statistics for biologists contains articles on many of the points above.

Software and code

Policy information about availability of computer code

Data collection	Imaris
Data analysis	3D image stacks were preprocessed using ImagJ2 . Image analysis was performed using Imaris (Version 9.6.0- 10.0, Oxford Instruments, Abington, UK).<br>Statistical analysis was done using the Prism10 Software (GraphPad, LaJolla, CA).<br>PCA was performed using OriginPro (OriginLab, Northhampton, MA).<br>Systems of non-linear equations were solved using Matlab (version R2021a, MathWorks, Natick, MA).

For manuscripts utilizing custom algorithms or software that are central to the research but not yet described in published literature, software must be made available to editors and reviewers. We strongly encourage code deposition in a community repository (e.g. GitHub). See the Nature Portfolio guidelines for submitting code & software for further information.

Data

Policy information about availability of data

All manuscripts must include a data availability statement. This statement should provide the following information, where applicable:

- Accession codes, unique identifiers, or web links for publicly available datasets
- A description of any restrictions on data availability
- For clinical datasets or third party data, please ensure that the statement adheres to our policy

The authors declare that the data supporting the findings of this study are available within the article and its Supplementary Information. Source data are provided with this paper.

Research involving human participants, their data, or biological material

Policy information about studies with human participants or human data. See also policy information about sex, gender (identity/presentation), and sexual orientation and race, ethnicity and racism.

Reporting on sex and gender	N/A
Reporting on race, ethnicity, or other socially relevant groupings	N/A
Population characteristics	N/A
Recruitment	N/A
Ethics oversight	N/A

Note that full information on the approval of the study protocol must also be provided in the manuscript.

Field-specific reporting

Please select the one below that is the best fit for your research. If you are not sure, read the appropriate sections before making your selection.

☒ Life sciences ☐ Behavioural & social sciences ☐ Ecological, evolutionary & environmental sciences

For a reference copy of the document with all sections, see nature.com/documents/nr-reporting-summary-flat.pdf

Life sciences study design

All studies must disclose on these points even when the disclosure is negative.

Sample size	We describe the development of novel methods. Therefore, predetermination of suitable samples sizes was not feasible as no prior data (e.g. regarding effect variations) was available. We demonstrate that intraasay variations were small, allowing for definite effect validations even with small sample sizes (n = 3-4)
Data exclusions	No data was excluded
Replication	Key experiments were repeatedly replicated. Demonstrating interassay reproducibility is a central element of the manuscript. Results from replications are therefore prominently shown in the manuscript (e.g. Extended Data Figure S5a,b)
Randomization	For tumor-treatment studies, engrafted tumor-bearing mice were randomly assigned to the different treatment groups just prior to the start of treatment.
Blinding	Blinding was not relevant for this study: i) the study was not hypothesis driven. Thus, a particular outcome was not favorable for the project. ii) both data acquisition (by LSFM) and data analysis (using a commercial software package) involves complex, standardized methods. iii) full samples (TMOs) were evaluated, a selection by an researcher (e.g. selection of a specific "field of view", a source for bias) was not necessary.

Reporting for specific materials, systems and methods

We require information from authors about some types of materials, experimental systems and methods used in many studies. Here, indicate whether each material, system or method listed is relevant to your study. If you are not sure if a list item applies to your research, read the appropriate section before selecting a response.

Materials & experimental systems

n/a	Involved in the study
☐	☒ Antibodies
☐	☒ Eukaryotic cell lines
☒	☐ Palaeontology and archaeology
☐	☒ Animals and other organisms
☒	☐ Clinical data
☒	☐ Dual use research of concern
☒	☐ Plants

Methods

n/a	Involved in the study
☒	☐ ChIP-seq
☐	☒ Flow cytometry
☒	☐ MRI-based neuroimaging

Antibodies

Antibodies used	Antibodies used for immunofluorescence staining: anti-CD31 (Dako, Mouse, Cat# M0823, RRID AB_2114471), anti-CD11b (SigmaAldrich, Rabbit, Cat# SAB5600105, RRID:AB_2910138), anti-Collagen IV (Bio-Rad, Rabbit, Cat# 2150-0140, RRID:AB_2082644), anti-Hif1a (Bethyl (Thermofisher), Rabbit, Cat# A300-286A, RRID:AB_2117114), Cleaved-Caspase 3 (Cell Signaling, Cat# 9661, RRID:AB_2341188). Secondary antibodies: goat anti-mouse-Cy5 (Jackson ImmunoResearch, Cat# 115-175-166, RRID:AB_2338714), goat anti-rabbit-AlexaFluor 750 (Thermofisher, Cat# A-21039, RRID:AB_2535710).<br>Antibodies used for FACS analysis: anti-ALDH1A1-PE (SinoBiological, Cat# 11388-MM03-P, RRID:AB_2860344) and anti-CD44-Alexa Fluor 700 (ThermoFisher, Cat# 56-0441-80, RRID:AB_494012).
Validation	The CD31-AB was validated by its ability to stain cord like structures (pseudovessels) formed by HUVEC. The CD11b-AB was validated by its ability to stain single cells in TMOs cultivated with THP-1 cells, while a positive staining was absent in TMOs that lacked THP-1 cells. The Col IV-AB was validated by ability to stain perivascular sheets in TMOs and in histological sections of murine tumors. Hif1a and Cleaved Caspase3 Antibodies were validated by western blot on human samples. Both ABs produced a single band in the appropriate MW range.<br>All antibodies have been validated for use on human samples by the manufactures:<br>CD31: https://www.agilent.com/en/product/immunohistochemistry/antibodies-controls/primary-antibodies/cd31-endothelial-cell-(concentrate)-76539<br>CD11b: https://www.sigmaaldrich.com/DE/de/product/sigma/sab5600105<br>Collagen IV: https://www.bio-rad-antibodies.com/polyclonal/human-collagen-iv-antibody-2150-0140.html<br>Cleaved Caspase-3: https://www.cellsignal.com/products/primary-antibodies/cleaved-caspase-3-asp175-antibody/9661?gclsrc=ds<br>Hif1a:https://www.thermofisher.com/antibody/product/HIF1-alpha-Antibody-Polyclonal/A300-286A

Eukaryotic cell lines

Policy information about cell lines and Sex and Gender in Research

Cell line source(s)	MCF7 (HTB-22), MDA-MB-435s (HTB-129), MDA-MB-231 (HTB-26), Sk-Br3 (HTB-30), ZR-75-1 (CRL-1500), MDA-MB-468 (HTB-132), and THP1 (TIB-202) cells were obtained from ATCC. AT3 cells were obtained from SigmaAldrich (Catalog # SCC178). HUVEC-2 were obtained from SigmaAldrich (Catalog # C-12208). NHDF were purchased from Lonza (Catalog # CC-2509).
Authentication	MCF7, MDA-MB-231 and MDA-435s were authenticated by the cell line authentication service of the German Collection of Microorganisms and Cell Cultures GmbH (DSMZ, Braunschweig, Germany). The other cell lines were acquired just before start of the studies.<br>Morphology, growth rate and appearance of the cells were monitored and matched throughout the experiments reporter characteristics of the respective cell line.
Mycoplasma contamination	Cell lines were routinely tested at least every six months for mycoplasma contamination (Mycoplasma PCR detection Kit, Catalog # G238, ABM, Richmond, BC, Canada) and found negative for mycoplasma contamination throughout the experiments.
Commonly misidentified lines (See ICLAC register)	The cell lines used are not commonly misidentified according to ICLAC

Animals and other research organisms

Policy information about studies involving animals; ARRIVE guidelines recommended for reporting animal research, and Sex and Gender in Research

Laboratory animals	Mus musculus, Strain C57BL/6J, female, 6-8 weeks
Wild animals	No wild animals were used in this study.

Reporting on sex	The research focused on breast cancer. For the study in an murine breast cancer model only female C57BL/6J mice were used.
Field-collected samples	No field-collected samples were used in this study.
Ethics oversight	The responsible regulatory body, the "Regierung von Unterfranken" (Regional administration for the district of lower franconia) approved and supervised the study. The statement is included in the manuscript.

Note that full information on the approval of the study protocol must also be provided in the manuscript.

Plants

Seed stocks	N/A
Novel plant genotypes	N/A
Authentication	N/A

Flow Cytometry

Plots

Confirm that:

- [x] The axis labels state the marker and fluorochrome used (e.g. CD4-FITC).
- [x] The axis scales are clearly visible. Include numbers along axes only for bottom left plot of group (a 'group' is an analysis of identical markers).
- [x] All plots are contour plots with outliers or pseudocolor plots.
- [x] A numerical value for number of cells or percentage (with statistics) is provided.

Methodology

Sample preparation	10-20 pooled TMOs were washed with PBS and digested for 10 min in trypsin/EDTA in PBS at 37°C then for 20 min in 10 mg/mL collagenase I (Worthington Biochemical Co., Lakewood, NJ) in DMEM (w/o FBS or P/S) at 37°C.
Instrument	FACSCelesta (BD Bioscience)
Software	Data was analyzed with FlowJo 10.0 (BD Bioscience).
Cell population abundance	N/A
Gating strategy	The gating strategy is shown in Extended Data Figure S9d: Events were gated by FSC-A vs. SSC-A for probable cells, then by FSC-A vs. FSC-H for single cell events. Single cells were gated for negativity in a Live/Dead staining (Thermofisher Cat# L34975). Live cells were than gated for GFP+ (=tumor cells), and tumor cells evaluated for strength of CD44 (Alexa Fluor 700) and ALDH1A1 (PE) staining.

- [x] Tick this box to confirm that a figure exemplifying the gating strategy is provided in the Supplementary Information.
