# Supplementary material for: A vascularized breast cancer spheroid platform for the ranked evaluation of tumor microenvironment-targeted drugs by light sheet fluorescence microscopy
Source: Nat Commun. 2024 Apr 27;15:3599. doi: 10.1038/s41467-024-48010-z (PMC11055956; doi:10.1038/s41467-024-48010-z)
Supplement: Supplementary file 4 — Description of Additional Supplementary Files [file 41467_2024_48010_MOESM4_ESM.pdf]

Supplementary Movie 1: Cellular compartmentalization in a Sk-Br-3-based VTS. The VTS was harvested and stained for CD31 (PV, red) and CD11b (yellow) after nine days of cultivation. Sk-Br-3 tumor cells expressed GFP (green outlines, after compartmentalization), and fibroblasts were identified by DsRed expression (blue outlines, after compartmentalization).

Supplementary Movie 2: Segmentation of the PV. Original CD31-fluorescence signal (red) and PV surface segmented from this signal (yellow, transparent and solid). MCF7-based VTS. 3D grid spacing: 50 µm.

Supplementary Movie 3: Tracing of the PV. Original CD31-fluorescence signal (red) and PVs traced along this signal (colored according to PV mean diameter). MCF7-based VTS. 3D grid spacing: 50 µm.

Supplementary Movie 4: Segmentation of TCs. Original GFP-fluorescence signal (green) and TC surfaces segmented in rendered from this signal (transparent yellow). MDA-MB-468-based VTS. 3D grid spacing: 50 µm.

Supplementary Movie 5: Time course of PV remodeling during cultivation. MDA-MB-231-based VTSs were harvested and processed after 3, 6, 9, and 12 days of cultivation, respectively.

Supplementary Movie 6: Distribution of Fibroblasts (blue) and MDA-MB-435s tumor cells (green) with respect to CD31 IF-stained PV (red). Coronal cut through a VTS harvested and processed after nine days of cultivation.

Supplementary Movie 7: Density and distribution of CD11b$^+$ macrophages in an MCF7-based VTS. CD31$^+$ PV in red. CD11b$^+$ macrophages in yellow.

Supplementary Movie 8: Density and distribution of CD11b$^+$ macrophages in a Sk-Br-3-based VTS. CD31$^+$ PV in red. CD11b$^+$ macrophages in yellow.

Supplementary Movie 9: Col IV-sleeve in MDA-MB-435s- and MCF7-based VTS. Rendered CD31$^+$- PVs are shown in red, Col IV in blue.

Supplementary Movie 10: Structures within an MDA-MB-435s-based VTS cultivated at 20% O_2. CD31$^+$-PVs in red, fibroblasts as transparent blue structures, and tumor cells in green.

Supplementary Movie 11: Structures within an MDA-MB-435s-based VTS cultivated at 2% O_2. CD31$^+$-PVs in red, fibroblasts as transparent blue structures, and tumor cells in green.

Supplementary Movie 12: Structure of an MDA-MB-435s-based VTS treated with a control substance. CD31$^+$-PVs in red, the position of tumor cells is shown as green orbs of 10 µm diameter.

Supplementary Movie 13: Structure of an MDA-MB-435s-based VTS treated with axitinib. CD31$^+$-PVs in red, the position of tumor cells is shown as green orbs of 10 µm diameter.

Supplementary Movie 14: Structure of an MDA-MB-435s-based VTS treated with LDC 1267. $CD31^+$-PVs in red, the position of tumor cells is shown as green (outside VTS shell) or yellow (inside VTS shell) orbs of 10 µm diameter.

Supplementary Movie 15: Structure of an MDA-MB-435s-based VTS treated with DesOH LY-411575. $CD31^+$-PVs in red, the position of tumor cells is shown as green (outside VTS shell) or yellow (inside VTS shell) orbs of 10 µm diameter.

Supplementary Movie 16: Hypoxic cells within an MDA-MB-468-based VTS treated with a control substance. $CD31^+$-PVs in red, the position of $Hif1a^+$-cells shown as turquoise orbs of 5 µm diameter.

Supplementary Movie 17: Hypoxic cells within an MDA-MB-468-based VTS treated with a UK 383367. $CD31^+$-PVs in red, the position of $Hif1a^+$-cells shown as turquoise orbs of 5 µm diameter.
